# DMoVGPE: predicting gut microbial associated metabolites profiles with deep mixture of variational Gaussian Process experts

**DOI:** 10.1186/s12859-025-06110-7

**Published:** 2025-03-27

**Authors:** Qinghui Weng, Mingyi Hu, Guohao Peng, Jinlin Zhu

**Affiliations:** 1 The State Key Laboratory of Food Science and Resources, Jiangnan University, Wuxi, 214122 Jiangsu People’s Republic of China; 2 The School of Artificial Intelligence and Computer Science, Jiangnan University, Wuxi, 214122 Jiangsu People’s Republic of China; 3 The School of Electrical and Electronic Engineering, Nanyang Technological University, Singapore, 639798 Singapore

**Keywords:** Gut microbiome, Metabolite prediction, Mixture of experts, Gaussian process regression, Automatic relevance determination

## Abstract

**Background:**

Understanding the metabolic activities of the gut microbiome is vital for deciphering its impact on human health. While direct measurement of these metabolites through metabolomics is effective, it is often expensive and time-consuming. In contrast, microbial composition data obtained through sequencing is more accessible, making it a promising resource for predicting metabolite profiles. However, current computational models frequently face challenges related to limited prediction accuracy, generalizability, and interpretability.

**Method:**

Here, we present the Deep Mixture of Variational Gaussian Process Experts (DMoVGPE) model, designed to overcome these issues. DMoVGPE utilizes a dynamic gating mechanism, implemented through a neural network with fully connected layers and dropout for regularization, to select the most relevant Gaussian Process experts. During training, the gating network refines expert selection, dynamically adjusting their contribution based on the input features. The model also incorporates an Automatic Relevance Determination (ARD) mechanism, which assigns relevance scores to microbial features by evaluating their predictive power. Features linked to metabolite profiles are given smaller length scales to increase their influence, while irrelevant features are down-weighted through larger length scales, improving both prediction accuracy and interpretability.

**Conclusions:**

Through extensive evaluations on various datasets, DMoVGPE consistently achieves higher prediction performance than existing models. Furthermore, our model reveals significant associations between specific microbial taxa and metabolites, aligning well with findings from existing studies. These results highlight DMoVGPE’s potential to provide accurate predictions and to uncover biologically meaningful relationships, paving the way for its application in disease research and personalized healthcare strategies.

**Supplementary Information:**

The online version contains supplementary material available at 10.1186/s12859-025-06110-7.

## Introduction

The microbiome and metabolite are increasingly recognized as key players in regulating human health. Microbial communities interact closely with human host through the production, metabolism, and modification of small molecules, which play essential roles in diverse biological processes [1, 2]. These microbial metabolites have been implicated in a wide range of physiological functions, from immune regulation to metabolic homeostasis and neurocommunication [3, 4]. For example, microbiome-derived metabolites such as trimethylamine-N-oxide (TMAO) have been linked to cardiometabolic diseases [5], while short-chain fatty acids (SCFAs) influence immune regulation and metabolism [6, 7]. Therefore, understanding the extensive repertoire of metabolites produced by the microbiome is crucial for unraveling the complex interplay between microbes and their human host, which could lead to new insights into disease mechanisms and therapeutic strategies. [8–10].

In recent years, metabolomics has seen rapid advancements [11]. Targeted metabolomics enables highly precise quantification of known metabolites, but its limited coverage restricts the discovery of unknown metabolites [12]. On the other hand, non-targeted metabolomics offers the advantage of broad coverage, detecting all measurable metabolites, making it ideal for discovering novel biomarkers. However, it also presents challenges, such as high resource consumption [13]. In addition, the complexity of microbial communities and their diverse metabolic outputs adds further challenges to fully exploring and understanding these interactions. To address these limitations, along with the substantial increase in human gut microbiome and metabolic data driven by the development of high-throughput sequencing technologies and the rapid advancement of machine learning (ML) techniques, scientists in recent years have been exploring and developing computational methods to predict metabolic features based on microbiome data [14–17]. ML algorithms are particularly effective at identifying complex patterns and relationships between the microbiome and its associated metabolites, which are challenging to capture using traditional experimental methods. Several ML models have been developed to map extensive genomic features to metabolites, falling into two main categories: linear regression models and neural network models. In the realm of linear regression, Himel Mallick et al. introduced the MelonnPan method, which used elastic network regression to predict metabolite abundance from taxonomic or functional features derived from metagenomics data [18]. While MelonnPan is computationally efficient and interpretable, its linear assumption limits its ability to capture complex nonlinear relationships between microbes and metabolites, which are prevalent in real biological systems. Additionally, MelonnPan does not account for shared information across metabolomics features, potentially missing important interactions that could improve prediction accuracy. The SparseNED model proposed by Vuong Le et al. employed an encoder-decoder structure with non-negative weights, which, although improving interpretability, restricted the model's learning capacity by enforcing sparsity and non-negativity constraints. The sparsity constraint limits the number of active connections in the network, potentially omitting important interactions between microbes and metabolites, while the non-negative weight constraint hinders the model's ability to capture negative correlations, which are common in microbial-metabolite interactions. [19]. Similarly, the Microbiome-Metabolome Network (MiMeNet) proposed by Derek Reiman et al. used a multilayer perceptron but faced challenges in interpretability due to its "black-box" nature. MiMeNet can compute feature attribution scores to quantify the contribution of microbes to metabolite predictions. However, the nonlinear transformations in the hidden layers obscure the relationship between input features and output predictions. This limits the model's interpretability despite its predictive capabilities. Furthermore, MiMeNet lacks the ability to model dynamic or time-dependent interactions between microbes and metabolites, which are often crucial for understanding metabolic processes in real biological systems [20]. Finally, the metabolomic profile predictor using neural ordinary differential equations (mNODE) model designed by Wang et al. demonstrated improved performance with advanced neural ordinary differential equations [21], but its generalizability was inconsistent across different datasets. This inconsistency stems from its high computational complexity arising from the need to solve ordinary differential equations during training and inference, which significantly increases computational overhead. mNODE's performance heavily depends on the quality and quantity of training data. Its reliance on ODEs may not align with the static nature of some microbiome-metabolome datasets. This reduces its applicability in scenarios requiring rapid or large-scale predictions.. These predictive models offer an alternative to traditional metabolomic analysis, allowing researchers to infer metabolite production and metabolic potential without direct biochemical measurements.

Despite the progress made by these predictive models, several challenges remain unaddressed, including the need for improved generalizability across diverse datasets, better handling of uncertainty, and enhanced biological interpretability. Furthermore, existing approaches often lack the capacity to adapt to the heterogeneity of microbiome-metabolite interactions across varying health conditions. To bridge these gaps, this paper introduces the Deep Mixture of Variational Gaussian Process Experts (DMoVGPE) model, which builds upon the strengths of previous methods while addressing their limitations. By integrating the Mixture of Experts (MoE) framework and Variational Gaussian Process Regression (VGPR), DMoVGPE represents a significant advancement in modeling the intricate relationships between gut microbiota and host metabolites. The MoE framework offers a statistically principled approach that weights each expert’s prediction through a gating network. This network probabilistically assigns different regions of the input space to specific experts. A key advantage of the MoE framework is its flexibility. By enabling different experts to focus on distinct data subsets, the model can capture a variety of behaviors, such as varying smoothness, heterogeneity, and even discontinuities within the input space. First, during the training process, we train different VGPR expert models in DMoVGPE using sample sets from various health conditions, allowing each expert to precisely capture the microbiome-metabolite associations specific to its assigned health condition. One of the key advantages of VGPR in this context is its ability to provide accurate predictions while accounting for the uncertainty in the model. As a Bayesian approach, VGPR offers strong interpretability, enabling the understanding of both the model's predictions and the underlying data relationships. Additionally, VGPR is particularly adept at modeling the correlations between multiple outputs, making it a powerful tool for understanding the complex, multi-dimensional nature of microbiome and metabolite data. DMoVGPE leverages a deep neural network (DNN) as the gating mechanism to dynamically assign expert model weights during the prediction of new samples, thereby enabling accurate predictions of metabolic abundances. Comparative experiments further demonstrate that our modeling approach enhances the prediction accuracy of microbiome-metabolite relationships across different health states. Additionally, the model incorporates an Automatic Relevance Determination (ARD) mechanism, which can identify the contribution strength of all microbial taxa features in the model input to the predicted metabolites. This not only improves model performance but also provides deeper insights into the underlying relationships between gut microbiota and metabolites. Experimental results demonstrate that DMoVGPE excels in handling multidimensional biological data and predicting microbiome-metabolite relationships, outperforming other existing methods [18–21], offering a powerful tool for advancing our understanding of the complex interactions between the gut microbiome and host metabolism. In summary, the main contributions of this paper are listed as below:

1. A DMoVGPE framework is introduced for predicting gut microbiome-associated metabolites, combining the strengths of MoE and VGPR to handle the multidimensional and nonlinear nature of microbiome data. By partitioning the dataset into subgroups, such as disease and healthy states, each expert is trained on a specific dataset, allowing the model to better capture microbiome features under varying conditions and enhancing prediction accuracy for complex biological relationships.
2. An ARD mechanism is utilized to identify the microbial taxa most significantly associated with metabolites in various health states. This feature improves the biological interpretability of the expert model predictions, aiding in further biological research and discovery.
3. DMoVGPE demonstrates strong predictive performance across 14 datasets, excelling in multiple evaluation metrics, underscoring its reliability and effectiveness in microbiome research.

## Methods

### Datasets

In our experimental study, a total of 14 publicly available human gut microbial metabolite datasets were included, sourced from the curated repository maintained by the Borenstein Lab. These datasets can be accessed at: https://github.com/borenstein-lab/microbiome-metabolome-curated-data/tree/main/data/processed_data [22]. Each dataset contains paired metagenomic and metabolomic feature information, providing a comprehensive resource for studying microbial-metabolite interactions. The datasets were collected from diverse cohorts, encompassing individuals with varying health conditions, dietary habits, and geographic backgrounds. For example, some datasets include samples from individuals with inflammatory bowel disease (IBD), obesity, or other metabolic disorders, while BIO_ML dataset represents healthy controls. This diversity in sample sources ensures a broad representation of human gut microbial and metabolic profiles, enhancing the generalizability of our findings and enabling the exploration of microbial-metabolite relationships across different phenotypes.

To prepare the data for analysis, we performed several preprocessing steps. First, we removed microbial and metabolite features with more than 50% of their abundance values equal to zero. This step was necessary to reduce data sparsity and dimensionality, which can otherwise lead to overfitting or unreliable predictions. Next, we applied a zero-replacement function to substitute zero abundances with very small values, addressing numerical issues that arise during logarithmic transformations. Finally, we normalized the data using the central logarithmic ratio (clr) transformation, as follows [23]:

1 $${\text{clr}}(x_{i} ) = \log \left( {\frac{{[x_{{i1}} , \ldots ,x_{{iD}} ]}}{{\sqrt[D]{{\Pi _{{j = 1}}^{D} (x_{{ij}} )}}}}} \right)$$

where $x_{i}$ is the abundance data of microbial metabolites. Prior to the transformation, we also employed a zero-replacement function to substitute zero abundances with very small values to avoid numerical issues.

An overview of the datasets, including sample size, number of samples in each subdataset, number of microbial features and number of metabolite features, is provided in Table 1. The datasets were partitioned based on sample labels (e.g., health status or disease phenotype), allowing the MoE framework to assign samples to specialized experts, thereby improving the accuracy and interpretability of microbiome-metabolite profile predictions.

**Table 1:** Overview of the Datasets

Dataset	Sample size	Number of samples in each subdataset	Number of microbial features	Number of metabolite features
MFGM [24]	277	With.comp.food': 50, 'Without.comp.food': 92, 'Month12': 68,'Baseline': 67	30	81
ASD [25]	44	'Autistic': 23, 'Neurotypical': 21	107	53
INFANTS_DIABETES [26]	103	'Case': 66, 'Control': 37	33	184
PRETERMS [27]	75	'Septic': 23, 'Nec': 14, 'GI': 37, 'Control': 1	31	453
SINHA_CRC [28]	131	'CRC': 42, 'Control': 89	31	530
ADENOMAS [29]	240	'Adenoma': 102, 'Carcinoma': 36, 'Control': 102	55	462
BIO_ML [30]	164	'Healthy': 164	29	489
IBD_RELATIVES [31]	90	'UC': 10, 'CD': 26, 'Normal': 54	117	1052
IBS [32]	444	'C': 133, 'D': 172, 'H': 139	1186	43
CRC [33]	347	'Stage_I_II': 69, 'Stage_III_IV': 54, 'Stage_0': 27, 'HS': 30, 'MP': 40, 'Healthy': 127	2983	165
GC [34]	96	'Gastrectomy': 42, 'Healthy': 54	6441	144
ESRD [35]	287	'ESRD': 220, 'Control': 67	7492	276
IBD [36]	220	'UC': 76, 'CD': 88, 'Control': 56	5604	7156
IBDMDB [37]	382	'UC': 101, 'CD': 177, 'Control': 104	2875	81,867

## Variational Gaussian Process regression

VGPR are fundamental components in many statistical and machine learning models, providing a probabilistic framework for modeling unknown functions, which allows for uncertainty quantification in predictions [38]. In regression tasks, VGPR is frequently used as priors for unknown functions, $f:x \to y$, due to their nonparametric nature and mathematical tractability. A Gaussian process (GP) begins by assuming that the underlying function follows a prior distribution, which is then refined based on observed data. By evaluating the degree of correlation between the function and the observed samples, the GP identifies the function that best aligns with the characteristics of the data, thereby enabling more accurate predictions. Given a finite set of input points $X = \{ x_{1} , \ldots ,x_{N} \} \subset {\mathbb{R}}^{d}$, assuming the function follows a GP prior, we write:

2 $$f(x)\sim GP(m(x),k(x,x^{\prime}))$$

The GP is characterized by two key components: mean function $m:{\mathbb{R}}^{d} \to {\mathbb{R}}$, covariance function (kernel) $k:{\mathbb{R}}^{d} \times {\mathbb{R}}^{d} \to {\mathbb{R}}$, the mean and covariance functions are given by:

3 $$m({\varvec{x}})={\mathbb{E}}[f({\varvec{x}})]$$

4 $$k\left( {{\varvec{x}},{\varvec{x}}{\prime }} \right) = {\mathbb{E}}[\left( {f\left( {\varvec{x}} \right) - m\left( {\varvec{x}} \right)} \right)\left( {f\left( {{\varvec{x}}{\prime }} \right) - m\left( {{\varvec{x}}{\prime }} \right))^{T} } \right]$$

Here, $m(x)$, the mean function, is often set to zero for simplicity, and represents the expected value of the function across input points. The covariance function $k(x,x^{\prime})$, also known as the kernel, quantifies the correlation between different inputs and depends on the model design. We adopt the squared exponential (SE) kernel, also known as the radial basis function (RBF), due to its smoothness, flexibility, and infinite-dimensional nature. The SE kernel is defined as:

5 $$k(x,x^{\prime}) = \sigma^{2} \exp \left( { - \frac{1}{2}r^{2} } \right) = \sigma^{2} \exp \left( { - \frac{{\left\| {x - x^{\prime}} \right\|^{2} }}{{2l^{2} }}} \right)$$

where $r$ is the Euclidean distance between the input points, scaled by the lengthscales parameter $l$, $\sigma^{2}$ is the variance parameter.

While isotropic kernel functions, such as the SE kernel, provide a simple model for capturing relationships in the data, they assume that the correlation between input dimensions is uniform. This can be limiting, as different features may exhibit varying levels of importance and interaction. To address these limitations, GPR introduces ARD, wherein each input dimension can have distinct length scale parameters. Specifically, GPR employs a vectorized representation, using a diagonal matrix to denote the length scale parameters for each dimension. The diagonal elements of this matrix represent the squares of the length scales for each dimension. Consequently, the length scale of each dimension can take on different values, thereby enabling the ARD framework. Furthermore, utilizing the ARD kernel function provides the Gaussian process with an automatic feature selection capability. This allows the model to autonomously select features that are pertinent to the prediction task, thereby enhancing both interpretability and performance. The ARD kernel of the SE is represented as:

6 $$k(x,x^{\prime}) = \sigma^{2} \exp \left( { - \frac{1}{2}\sum\limits_{i = 1}^{d} {\frac{{(x_{i} - x^{\prime}_{i} )^{2} }}{{l_{i}^{2} }}} } \right)$$

By integrating this ARD kernel into the Bayesian model selection framework, we further optimized the length-scale parameters by maximizing the model evidence:

7 $$p(d_{{1:n_{d} }} |\psi ) = \int {p(} d_{{1:n_{d} }} |\phi )p(\phi |\psi )d\phi ,$$

where $p(d_{{1:n_{d} }} |\phi )$ is the likelihood function, and $p(\phi |\psi )$ is the parameterized ARD prior distribution. Through the optimization of hyperparameters $\psi (i.e.,l_{1} ,l_{2} , \ldots ,l_{D} )$, the model effectively reduces redundant features and identifies the key contributors, resulting in a more efficient and interpretable dimensionality reduction process.

For regression tasks involving noisy observations, we define the model as $y(x) = f(x) + \varepsilon$, where $\varepsilon$ represents observation noise and follows a normal distribution $\varepsilon \sim {\mathcal{N}}(0,\sigma_{s}^{2} )$. Incorporating a noise term accounts for discrepancies between the model and real-world data, improving prediction robustness. Given a set of training points $X = \{ x_{1} , \ldots ,x_{N} \}^{T}$ from the domain $\Omega^{d}$ and their corresponding outputs $y = \{ y(x_{1} ), \ldots ,y(x_{n} )\}^{T}$, we leverage the fact that a GP is a stochastic process, where any finite set of random variables follows a joint Gaussian distribution. For a new test point $x^{*}$, the relationship between the observations $y$ and the latent function $f(x^{*} )$ is described by the joint prior distribution of the GP, which follows a multivariate Gaussian form:

8 $$\left[ {\begin{array}{*{20}c} {\varvec{y}} \\ {f({\varvec{x}}^{*} )} \\ \end{array} } \right]\sim {\mathcal{N}}\left( {\left[ {\begin{array}{*{20}c} 0 \\ 0 \\ \end{array} } \right],\left[ {\begin{array}{*{20}c} {K(X,X) + \sigma_{s}^{2} I} & {K(X,{\varvec{x}}^{*} )} \\ {K({\varvec{x}}^{*} ,X)} & {k({\varvec{x}}^{*} ,{\varvec{x}}^{*} )} \\ \end{array} } \right]} \right)$$

This joint Gaussian distribution is the foundation for making predictions and quantifying uncertainty in GP-based regression models.

In a Gaussian process, the covariance matrix $K(X,X) \in {\mathbb{R}}^{n \times n}$ represents the covariance between the observed data points and is a symmetric, positive semi-definite matrix. Given the joint Gaussian prior distribution over the observed data ${\varvec{y}}$, we can derive the conditional distribution for the function at a new test point ${\varvec{x}}^{*}$, $f({\varvec{x}}^{*} )$, as:

9 $$f({\varvec{x}}^{*} )|X,{\varvec{y}},{\varvec{x}}^{*} \sim {\mathcal{N}}(\hat{f}({\varvec{x}}^{*} ),\sigma^{2} ({\varvec{x}}^{*} ))$$

where the predicted mean $\hat{f}({\varvec{x}}^{*} )$ and predicted variance $\sigma^{2} ({\varvec{x}}^{*} )$ are given by:

10 $$\hat{f}({\varvec{x}}^{*} ) = K(X,{\varvec{x}}^{*} )\left[ {K(X,X) + \sigma^{2} I} \right]^{ - 1} {\varvec{y}}$$

11 $$\sigma^{2} ({\varvec{x}}^{*} ) = k({\varvec{x}}^{*} ,{\varvec{x}}^{*} ) - K({\varvec{x}}^{*} ,X)(K(X,X) + \sigma^{2} I)^{ - 1} K(X,{\varvec{x}}^{*} )$$

To train the model and optimize the hyperparameters, we use Negative Log Marginal Likelihood (NLML), a standard method for fitting Gaussian processes. NLML quantifies how well the model fits the observed data and is minimized to adjust the model parameters, such as the kernel hyperparameters. The general form of the NLML is:

12 $${NLML = - log }p({\varvec{y}}|X,\theta ) = \frac{1}{2}{\varvec{y}}^{T} \left[ {K(X,X) + \sigma^{2} I} \right]^{ - 1} {\varvec{y}} + \frac{1}{2}{log}\left| {K(X,X) + \sigma^{2} I} \right| + \frac{n}{2}\log 2\pi$$

Despite its flexibility and broad applicability, VGPR faces several significant limitations, particularly in microbiome-metabolite studies. First, VGPR's computational inefficiency, due to the cubic time complexity of handling large covariance matrices, severely limits its scalability for high-dimensional, large-scale datasets. In addition, the inherent rigid structure is ill-equipped to capture the heterogeneity and local variations often observed in microbiome-metabolite relationships, where distinct subsets of data may exhibit varying patterns or degrees of smoothness. Finally, as dataset size and complexity increase, VGPR's predictive accuracy tends to decline, making it less suitable for modeling intricate, region-specific behaviors or discontinuities in large-scale studies. These challenges highlight the critical need for more scalable and flexible modeling techniques to address the demands of high-dimensional, heterogeneous datasets effectively.

## Deep mixture of variational Gaussian Process experts

To address the computational challenges of high-dimensional, large-scale microbiome-metabolite data, a common strategy involves partitioning the data and employing multiple Gaussian Process experts, each tasked with modeling a specific subset of the data. This decomposition improves scalability by allowing each expert to handle smaller covariance matrices, significantly reducing computational complexity. In this regard, we propose the MoE framework. Our MoE architecture dynamically selects a subset of experts for each input, which not only enhances computational efficiency but also retains the flexibility required to model the intricate relationships between microbiome features and metabolite concentrations.

The general MoE model assumes that outputs are independently generated from a mixture:

13 $$y_{i} |x_{i} \sim \sum\limits_{l = 1}^{L} {w_{l} (x_{i} ;\psi ){\text{N}}(} y_{i} |f(x_{i} ;\theta_{l} ),\sigma_{l}^{2} )$$

where $w_{l} ( \, \cdot \, ;\psi )$ is the gating network with parameters $\psi$,$f( \cdot \, ;\theta_{l} )$ is the regression function for the $l^{th}$ expert with parameters $\theta_{l}$, and $L$ is the number of experts. We introduce a novel MoE model that integrates the expressive power of DNNs with the probabilistic framework of GPs. While DNNs excel in flexibility and modeling complex patterns, they traditionally lack the built-in mechanism for uncertainty quantification that GPs provide. Recent research efforts have aimed to combine DNNs with GPs to leverage the advantages of both methodologies, as illustrated in studies by Iwata and Ghahramani [39], and Daskalakis et al. [40]. However, they often struggle to balance computational efficiency with the flexibility needed for modeling complex, heterogeneous data. In our approach, GP experts offer smooth, probabilistic reconstructions of the unknown regression function for each region, while DNNs serve as the gating network, effectively determining these regions based on the input data. This complementary integration allows the MoE model to capitalize on the strengths of DNNs for flexible representation and GPs for uncertainty estimation, resulting in a more robust predictive framework. In addition, we leverage the disease labels from the microbiome dataset to partition the samples into distinct subsets representing healthy and diseased states. Each GP expert is trained on these specialized subsets, ensuring that each expert is tailored to a specific health phenotype category. During inference, the gating network effectively routes each sample to the most appropriate expert, enabling the MoE model to make precise, condition-specific predictions. In contrast to VGPR, where the ARD mechanism focuses on population-wide characteristics, the ARD in DMoVGPE emphasizes disease-specific features, capturing the unique traits of different disease categories. By dynamically adjusting the length scales, our model identifies relevant features and suppresses irrelevant ones, offering an effective and interpretable method for feature selection in complex biological data analysis.

Building on this foundation, we can now focus on the specific implementation of DNNs as the gating network within the MoE model. DNNs are known for their universality in classification tasks [41]. DNNs flexibly determine the regions without making any rigid assumptions and without increasing the computational cost. Specifically, the gating network is defined by a feedforward DNN with a softmax output:

14 $$w_{l} (x;\psi ) = \frac{{\exp (h_{l} (x;\psi ))}}{{\sum\nolimits_{j = 1}^{L} {\exp (h_{j} (x;\psi ))} }}$$

where $h_{l}$ is the $l^{th}$ component of $h:{\mathbb{R}}^{d} \to {\mathbb{R}}^{L}$, defined by

15 $$h( \, \cdot \, ;\psi ) = \eta_{J} (\eta_{J - 1} ( \cdots \eta_{1} ( \, \cdot \, ;\psi_{1} ) \cdots ;\psi_{J - 1} );\psi_{J} ),$$

with $\eta_{j} :{\mathbb{R}}^{{d_{j - 1} }} \to {\mathbb{R}}^{{d_{j} }} (d_{0} = d,d_{J} = L)$ being the $j^{th}$ layer of a neural network. $\eta_{j} ( \, \cdot \, ;\psi_{j} ):x \mapsto \eta_{j} (x;\psi_{j} ) = {\text{RELU}}(A_{j} x + b_{j} )$, and ${\text{RELU}}(x) = \max \{ 0,x\}$ is the element-wise rectifier. $\psi_{j} = \{ A_{j} ,b_{j} \}$ comprises the weights $A_{j} \in {\mathbb{R}}^{{d_{j} \times d_{j - 1} }}$ and biases $b_{j} \in {\mathbb{R}}^{{d_{j} }}$ for level $j = 1, \ldots ,J$. $\psi = (\psi_{1} , \ldots ,\psi_{J} )$ collects all the parameters of the DNN. The final output of the MoE model is calculated as a weighted sum of predictions from all experts. Specifically, given $L$ experts, each producing a prediction $f(x_{i} ;\theta_{l} )$ for the input $x_{i}$, and the gating network assigns a weight $w_{l} (x_{i} ;\psi )$ to the $l$-th expert, the overall output can be expressed as:

16 $$\mu (x_{i} ) = \sum\limits_{l = 1}^{L} {w_{l} (x_{i} ;\psi } ) \cdot f(x_{i} ;\theta_{l} )$$

17 $$\sigma^{2} (x_{i} ) = \sum\limits_{l = 1}^{L} {w_{l} (x_{i} ;\psi } ) \cdot (\sigma_{l}^{2} + (f(x_{i} ;\theta_{l} ) - \mu (x_{i} ))^{2} )$$

where $\mu (x_{i} )$ represents the weighted mean of the predictions. As can be seen, $\sigma^{2} (x_{i} )$ accounts for both the inherent uncertainty of each expert and the variance introduced by deviations of individual predictions from the mean.

**Fig. 1:**
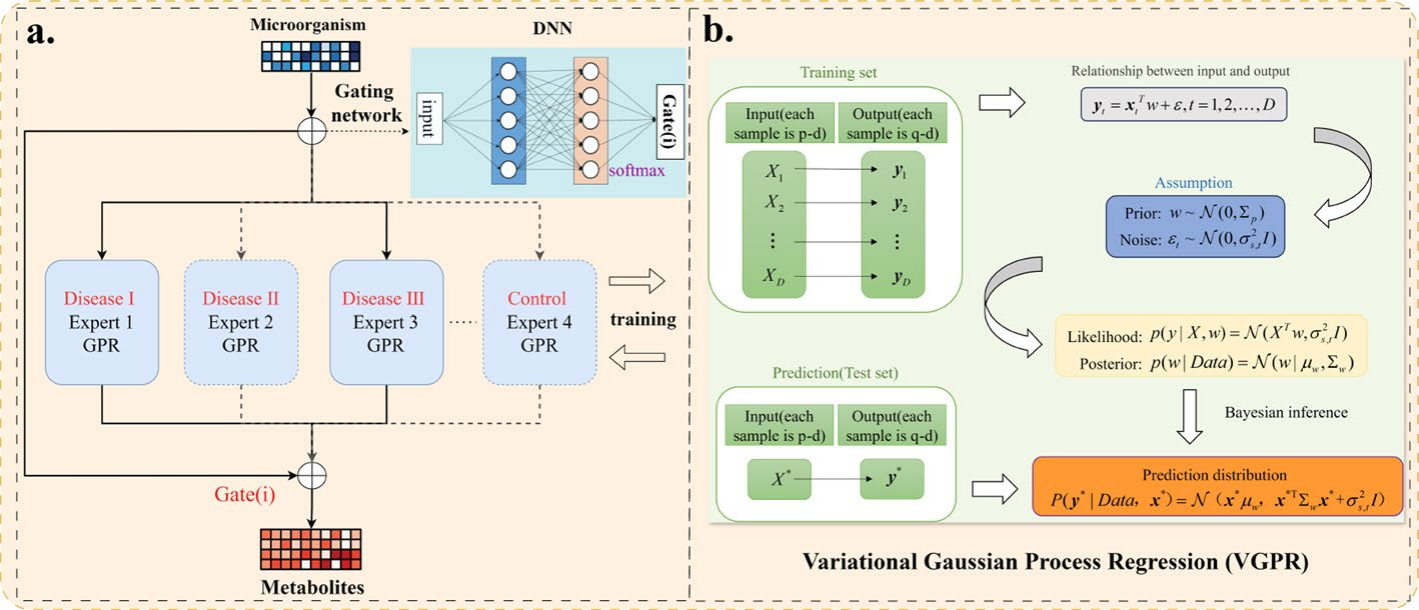
DMoVGPE model architecture diagram. Figure a. illustrates the overall architecture of the DMoVGPE model. A gating network, implemented as a neural network with a softmax activation function, assigns probabilities to multiple VGPR experts based on the input microbial data. Each expert is specialized for a specific disease state, such as Disease I, Disease II, Disease III, or Control. The gating network dynamically weights the predictions from these experts, enabling a weighted aggregation of their outputs for each input. This dynamic weighting mechanism ensures the model adapts to the input features, tailoring the prediction to disease-specific patterns. Figure b. provides a detailed view of the Variational Gaussian Process Regression (VGPR) process within each expert. Each VGPR expert uses a squared exponential kernel to compute the covariance matrix and performs Bayesian inference with microbial data. The VGPR outputs include a mean prediction and uncertainty for each metabolite feature. The model combines the outputs from the experts, weighted by the gating network, to produce a final prediction with confidence tailored to the input features

The model architecture diagram of DMoVGPE is shown in Fig. 1.

## Experiment and parameter setting

To construct the model, the gating network utilizes a neural architecture comprising two fully connected layers with ReLU activations, followed by a dropout layer to prevent overfitting. The network consists of a 128-unit dense layer, a 64-unit dense layer, and an output layer with a softmax activation function to generate expert selection probabilities. This design facilitates robust representation and dynamic weighting of the VGPR experts for optimal prediction. The samples were classified into distinct categories: Control, Disease I, Disease II, etc. To validate model performance, the fivefold cross-validation was employed. The model was optimized using the Adam optimizer with an exponential decay learning rate scheduler. The initial learning rate was set at 0.01, decaying by a factor of 0.95 every 500 steps to ensure smooth convergence during training. During training, the gating network dynamically adjusted the contribution of each expert based on the input features, with all experts remaining active but their influence varying according to the gating network’s output. As training progressed, the gating network refined the expert selection probabilities, assigning higher weights to the most relevant experts. This approach enables the model to leverage the strengths of multiple experts to capture the complex relationships between microbiome and metabolome data, thereby improving prediction accuracy across different disease states. In summary, the experts are trained to model disease-specific relationships, while the gating network learns to adjust the contributions of each expert dynamically for every input. Such an end-to-end training process enables mutual promotion between the local and global components, allowing both to improve simultaneously and enhancing the model’s ability to make accurate and interpretable predictions.

## Result

### Comparison of prediction performance

To rigorously evaluate the performance of DMoVGPE in metabolite prediction, we conducted a comparative analysis with several state-of-the-art methods: MelonnPan, SparseNED, MiMeNet, mNODE, as well as the expert model VGPR. All models were trained on the same preprocessed dataset, and their performance was assessed using Spearman's rank correlation coefficient (SCC), which quantifies the strength and direction of association between predicted and observed metabolite abundances [42].

For each sample, the SCC between the predicted and true metabolite abundances was computed, and the mean SCC across all samples provided an aggregate measure of model accuracy. To ensure consistency with previous work, we focus on the top ten ranked metabolites to assess the model's performance. The results are presented as mean values ± standard deviation, as shown in Table 2, with the best-performing method for each dataset highlighted in bold.

**Table 2:** Results of Benchmarking Models

Dataset	MelonnPan	SparseNED	MiMeNet	mNODE	VGPR	DMoVGPE
MFGM	0.546 ± 0.031	0.490 ± 0.040	0.546 ± 0.005	0.561 ± 0.095	0.525 ± 0.043	**0.566 ± 0.042**
ASD	0.583 ± 0.029	0.520 ± 0.082	0.406 ± 0.028	0.696 ± 0.072	0.620 ± 0.080	**0.703 ± 0.104**
INFANTS_DIABETES	0.680 ± 0.056	0.714 ± 0.016	0.577 ± 0.021	0.682 ± 0.038	0.720 ± 0.047	**0.728 ± 0.044**
PRETERMS	0.715 ± 0.015	0.743 ± 0.037	0.685 ± 0.016	0.740 ± 0.106	0.785 ± 0.040	**0.797 ± 0.033**
SINHA_CRC	0.509 ± 0.027	0.562 ± 0.028	0.396 ± 0.017	0.524 ± 0.037	0.581 ± 0.035	**0.612 ± 0.059**
ADENOMAS	0.654 ± 0.042	0.531 ± 0.039	0.542 ± 0.005	0.624 ± 0.053	0.637 ± 0.043	**0.667 ± 0.058**
BIO_ML	0.711 ± 0.059	0.686 ± 0.033	0.735 ± 0.003	0.667 ± 0.089	0.722 ± 0.026	**0.749 ± 0.024**
IBD_RELATIVES	0.630 ± 0.027	0.756 ± 0.046	0.649 ± 0.008	0.703 ± 0.055	0.779 ± 0.057	**0.790 ± 0.016**
IBS	0.248 ± 0.034	0.265 ± 0.022	0.369 ± 0.006	0.441 ± 0.087	0.305 ± 0.053	**0.465 ± 0.051**
CRC	0.486 ± 0.036	0.500 ± 0.015	0.520 ± 0.007	0.408 ± 0.084	**0.575 ± 0.017**	0.555 ± 0.034
GC	0.312 ± 0.046	0.607 ± 0.035	0.615 ± 0.016	0.633 ± 0.067	**0.772 ± 0.043**	0.669 ± 0.063
ESRD	0.419 ± 0.046	0.599 ± 0.017	0.498 ± 0.011	0.565 ± 0.042	**0.645 ± 0.019**	0.634 ± 0.036
IBD	0.708 ± 0.023	0.847 ± 0.026	0.703 ± 0.016	0.827 ± 0.036	0.857 ± 0.025	**0.857 ± 0.027**
IBDMDB	0.635 ± 0.040	0.663 ± 0.022	0.551 ± 0.003	0.551 ± 0.046	0.666 ± 0.017	**0.674 ± 0.020**
Average	0.560	0.606	0.556	0.616	0.655	**0.676**

The results summarized in Table 2 indicate that the DMoVGPE model outperformed other prediction models across all datasets, ranking first in 14 datasets with an average prediction score of 0.676. This highlights the exceptional predictive performance of DMoVGPE. Deep learning models such as SparseNED and MiMeNet can capture complex patterns but often require extensive hyperparameter tuning and large amounts of data to avoid overfitting, which limits their generalizability, especially with limited sample sizes or high noise. MelonnPan, on the other hand, models each metabolite independently, overlooking correlations among metabolites, while the mNODE model uses time-series data, which can impair its generalization ability. In contrast, the DMoVGPE model effectively captures heterogeneous relationships by using multiple Gaussian Process experts, each tailored to a specific disease state. VGPR was chosen as the base model for each expert within the mixture-of-experts framework due to its strong predictive capabilities, providing a robust foundation for capturing complex, disease-specific relationships when combined with the gating network. These results underscore DMoVGPE’s effectiveness in modeling the intricate relationships inherent in microbiome and metabolome data, as well as its capacity to generalize across diverse disease states.

Despite this overall success, Table 2 reveals that the DMoVGPE model faced some challenges in the GC dataset, where it underperformed compared to the baseline VGPR model. This discrepancy can be attributed to the relatively small sample size of the GC dataset, with only 96 samples divided into "Gastrectomy" and "Healthy" groups. When stratified by disease state, each expert in the DMoVGPE model receives fewer samples, making it more difficult to capture disease-specific patterns effectively. Additionally, the high-dimensional nature of the GC dataset—featuring 6,441 microbial features—further complicates the modeling process. In cases like this, where sample size is limited and the data is high-dimensional, the DMoVGPE model may struggle to fully leverage its mixture-of-experts framework, while a single VGPR model can more easily capture general trends across all samples. To address these limitations, future research could explore several promising directions. One approach is to develop adaptive kernel functions tailored to specific phenotypes, enabling the model to better capture the distinct patterns inherent in each subset of data. Furthermore, integrating attention mechanisms into the gating network could enhance its ability to model complex relationships within the data, leading to more nuanced and accurate predictions. Finally, refining the mixture-of-experts framework by dynamically adjusting the number of experts or their specialization criteria could improve the model's adaptability to datasets with imbalanced or limited samples. While the GC dataset presented challenges, the overall results strongly suggest that the DMoVGPE model excels in most scenarios, particularly when data is rich and well-distributed across disease states. These potential improvements not only address the current limitations but also pave the way for broader applications of the DMoVGPE model in diverse microbiome studies.

Furthermore, we conducted a performance comparison between the DMoVGPE model and existing methods using three evaluation metrics: the mean Spearman correlation coefficient (SCC) for all predicted metabolites, the mean SCC for the top 50 predicted metabolites, and the number of metabolites with an SCC greater than 0.5. MelonnPan is excluded from the comparison because it only outputs well-predicted metabolites (SCC > 0.3).

**Fig. 2:**
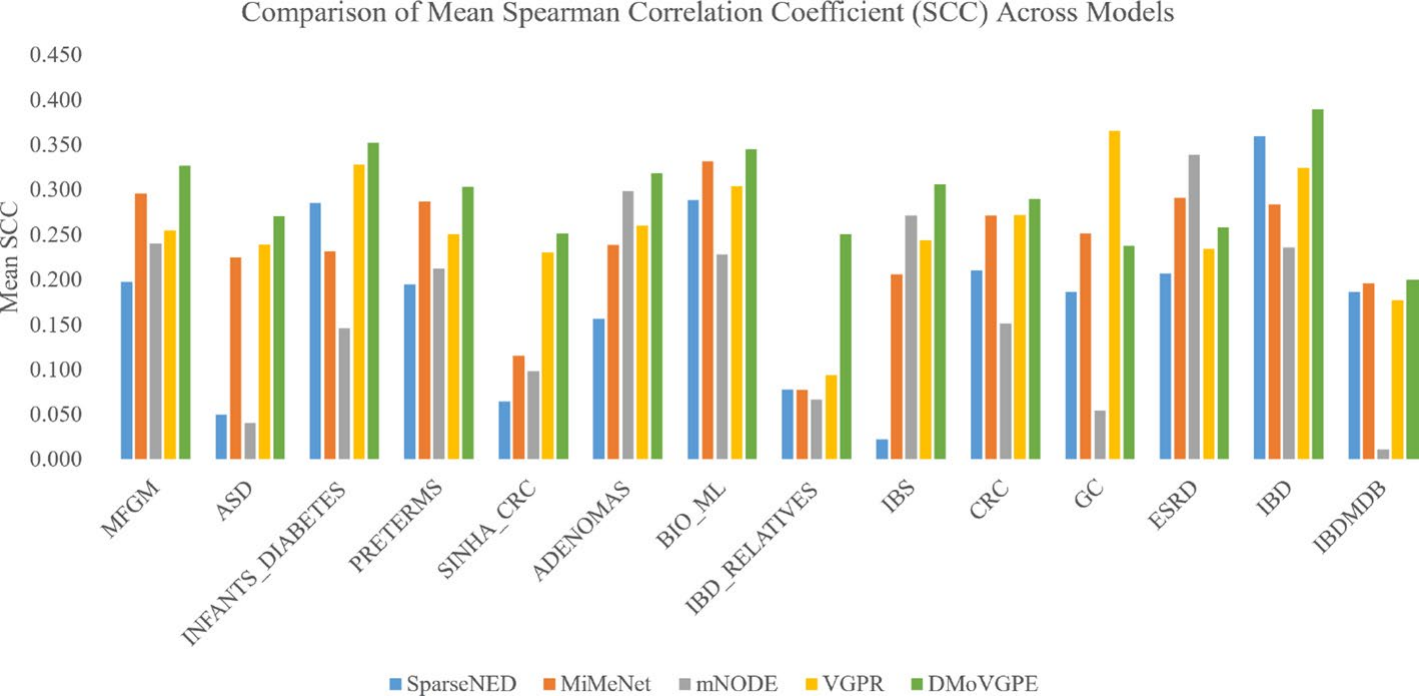
All metabolites predict SCC mean results. This figure illustrates the comparison of the mean Spearman correlation coefficient (SCC) predictions for all metabolites between DMoVGPE and other methods (SparseNED, MiMeNet, mNODE, VGPR) across 14 datasets

**Fig. 3:**
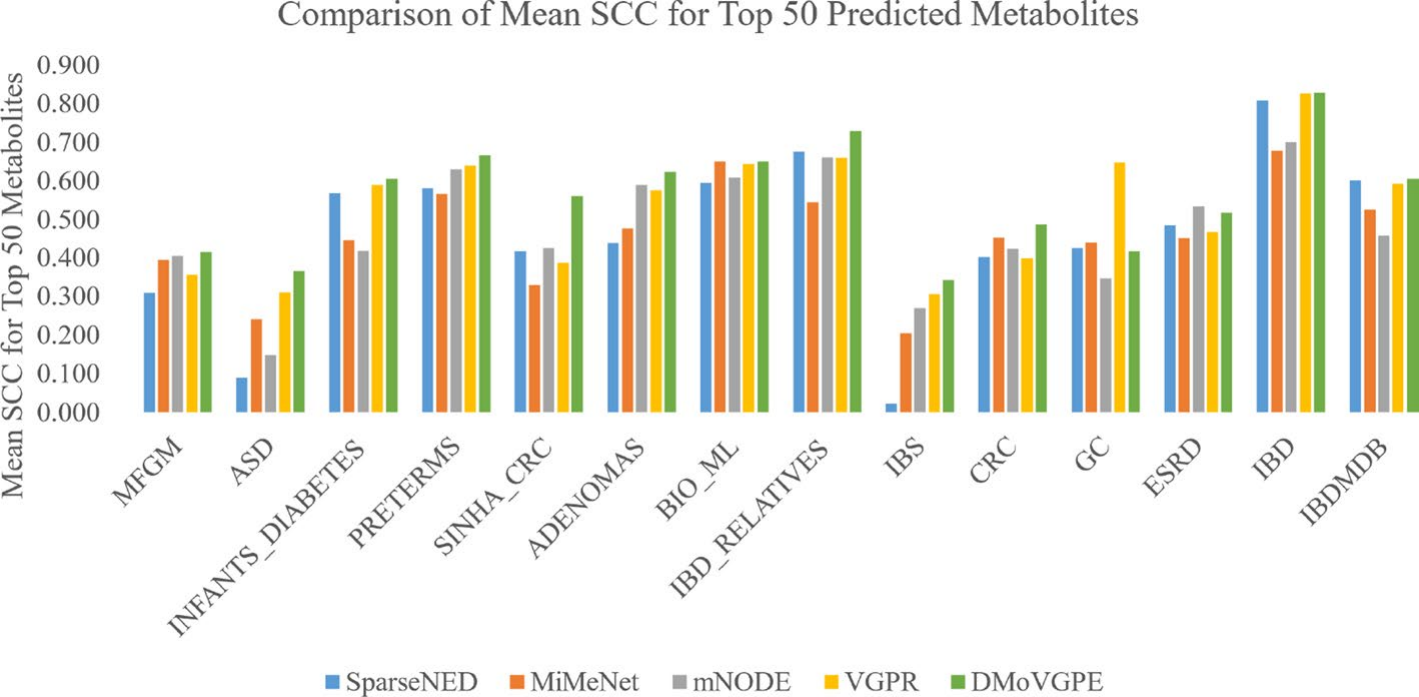
Prediction of SCC mean value of the top 50 metabolites. This chart presents a comparison of the mean Spearman correlation coefficient (SCC) predictions for the top 50 metabolites between DMoVGPE and SparseNED, MiMeNet, mNODE, VGPR across 14 datasets

**Fig. 4:**
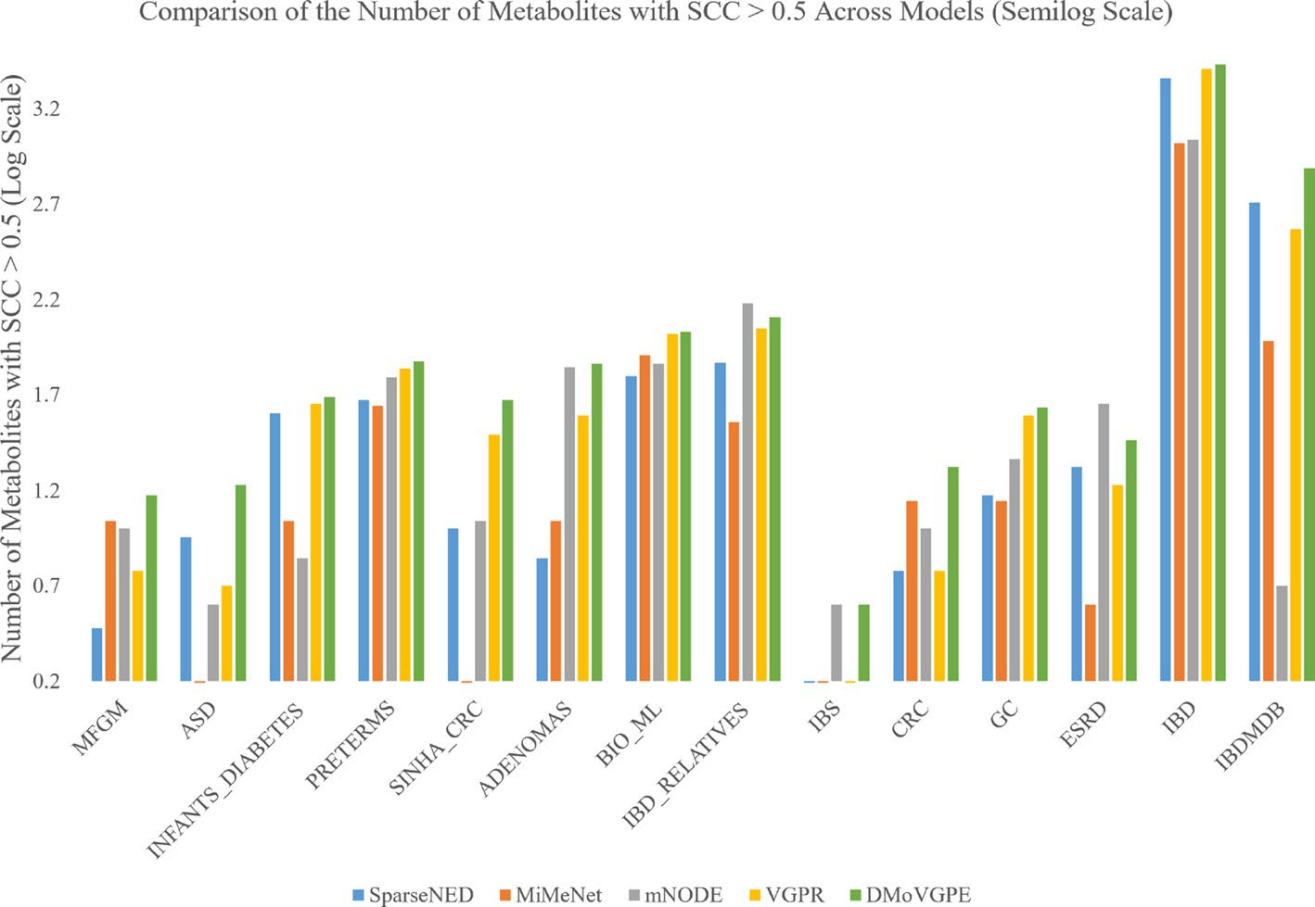
Predicting the number of metabolites with SCC > 0.5. To ensure consistent scaling across datasets and models, we applied a log10 transformation (adding 1 to avoid log(0)). This transformation standardizes the output while retaining the relative differences between the predictions of various models. Each bar in the figure represents the log-transformed number of metabolites predicted by the respective model for a given dataset with a SCC greater than 0.5

As shown in Fig. 2, our DMoVGPE model achieves the highest mean SCC for all predicted metabolites across 12 datasets. Similarly, Fig. 3 illustrates that DMoVGPE performs best in terms of the mean SCC for the top 50 predicted metabolites on 12 datasets. Figure 4 further demonstrates that DMoVGPE ranks first in the number of metabolites with an SCC greater than 0.5 on 11 datasets. This indicates that the DMoVGPE model consistently outperforms other methods across multiple metrics, highlighting its robustness and superior predictive power for microbiome data. Achieving the highest mean SCC across most datasets suggests that DMoVGPE provides more accurate predictions overall. Additionally, the model’s strong performance in predicting the top 50 metabolites and in the number of metabolites with SCC > 0.5 underscores its effectiveness at identifying highly relevant metabolites, making it a reliable tool for detailed metabolite prediction in microbiome research.

**Fig. 5:**
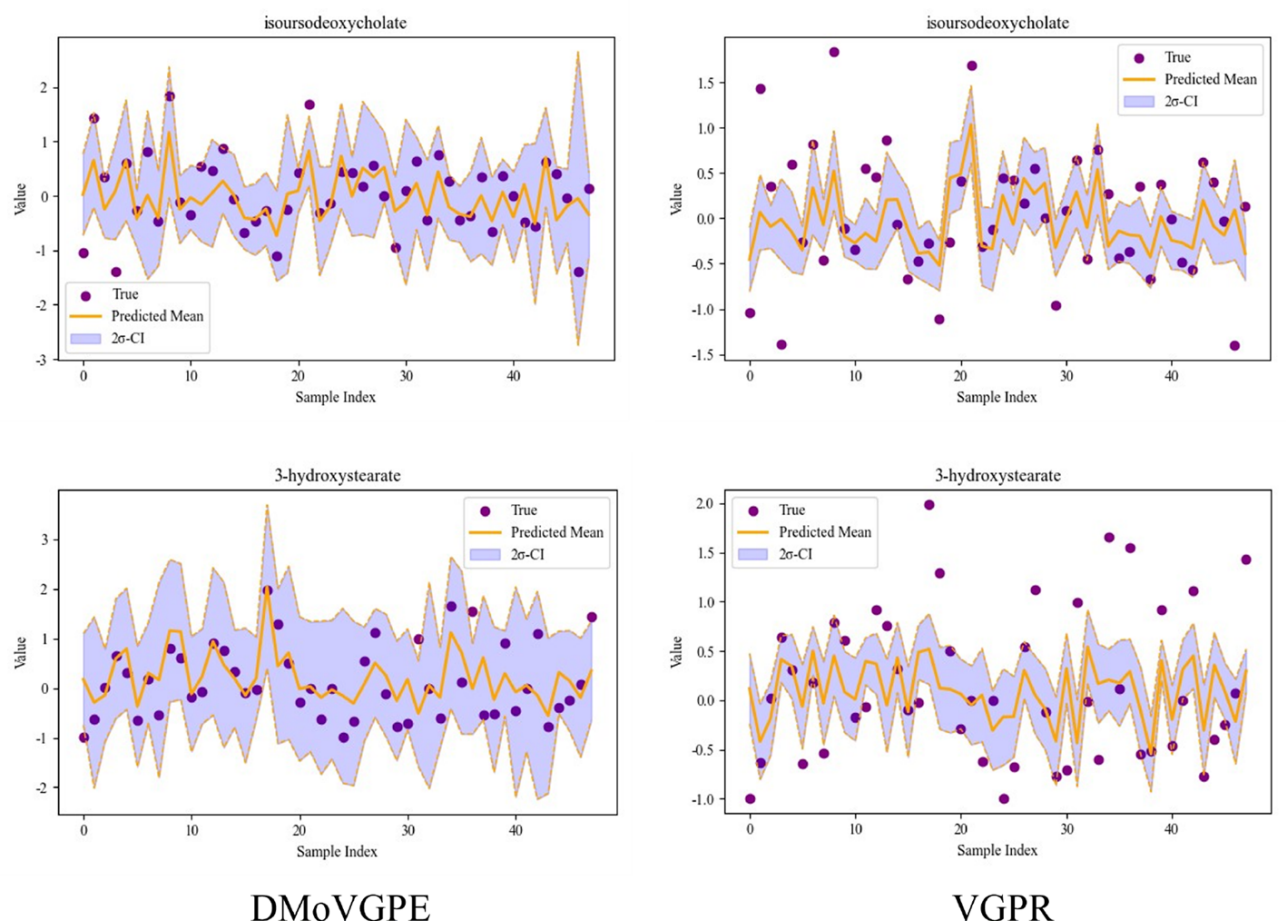
Predictive performance and uncertainty quantification of DMoVGPE on key metabolites from the ADENOMAS dataset. The purple dots represent the true values of the metabolites, while the orange line shows the predicted values from the DMoVGPE model. The purple shaded area indicates the 95% credible intervals (2σ-CI), which capture the uncertainty in the predictions

To further investigate the predictive capabilities of the DMoVGPE model with respect to VGPR, we use the ADENOMAS dataset as a case study to visualize the predicted results and associated confidence intervals on representative metabolites. As illustrated in Fig. 5, we focus on two metabolites—isoursodocycholate and 3-hydroxystearate—which were selected for their high predictive accuracy. The predicted means closely align with the true values, reflecting the model's high accuracy, while the well-calibrated 95% credible intervals capture the associated uncertainty, encompassing the true values even in regions of high variability. In contrast, the VGPR model demonstrates certain limitations. While VGPR provides reasonable predictions, its uncertainty estimates are often overly narrow, underrepresenting the true variability in the data. This shortcoming is particularly noticeable for metabolites with substantial variability, such as 3-hydroxystearate, where VGPR predictions deviate more significantly from the true values, reducing its robustness. The key strength of the DMoVGPE model lies in its ability to capture complex, nonlinear relationships in the data and to dynamically adapt to diverse patterns. The MoE framework enables specialized experts to handle distinct metabolic profiles, while the integration of ARD allows for effective feature prioritization and interpretation. These features collectively enable DMoVGPE to outperform VGPR, particularly in the analysis of intricate microbiome datasets. The model's flexibility, scalability, and robust uncertainty quantification position it as an effective tool for microbiome-metabolite prediction tasks.

## Comparative evaluation of expert allocation strategies

In this section, two comparative experiments have been conducted to evaluate the advantages and effectiveness of expert allocation strategy. The first approach used random data splits within each dataset, maintaining the same number of experts as in the original DMoVGPE model to introduce variability in each expert’s training data. This approach allowed us to verify whether disease-label-based splitting enhances the model's performance. The second approach divided the dataset based on disease health labels, then further split each label group in half to increase the number of experts and enhance specialization. This approach aimed to determine if additional experts improve predictive accuracy. The main objectives of this experiment is to (1) assess the effectiveness of disease-label-based or phenotype-based data splits and (2) examine how varying the number of experts impacts predictive accuracy.

As shown in Table 3, the DMoVGPE method with phenotype-based split consistently outperforms both the random data splitting and increased expert count (double experts) methods across the majority of analyzed datasets. Specifically, in datasets such as MFGM, ASD, and PRETERMS, DMoVGPE achieves higher mean performance scores of 0.566, 0.703, and 0.797, respectively. Furthermore, the results reveal that DMoVGPE exhibits lower standard deviations compared to other methods, indicating greater stability in its predictions. In the BIO_ML dataset, both DMoVGPE and the random partition method yield the same median score of 0.749, with identical standard deviations (± 0.024). This is because BIO_ML consists of data from healthy individuals with only one label; as a result, both the stratified cross-validation based on health labels and the random partitioning yield equivalent results. In contrast, the double experts method demonstrates greater variability in performance across datasets. For instance, the performance fluctuations observed in the ASD dataset (0.587 ± 0.085) highlight the limitations of the dual-expert approach in certain scenarios. Specifically, the ASD dataset consists of only 44 samples, and when split into two experts, each expert is left with fewer samples for training. This limited data availability results in insufficient training data for each expert, preventing the model from learning effectively and leading to poorer performance. In contrast, DMoVGPE, with half the number of experts, allows each expert to work with more data, which improves the model’s ability to capture patterns and results in better performance (Tables 4 and 5).

**Table 3:** Comparative Analysis

Dataset	Disease-Label-Based Split (DMoVGPE)	Random Data Splitting	Increased Expert Count (Double Experts)
MFGM	**0.566 ± 0.042**	0.556 ± 0.038	0.554 ± 0.038
ASD	**0.703 ± 0.104**	0.695 ± 0.089	0.587 ± 0.085
INFANTS_DIABETES	**0.728 ± 0.044**	0.668 ± 0.053	0.710 ± 0.062
PRETERMS	**0.797 ± 0.033**	0.778 ± 0.041	0.773 ± 0.036
SINHA_CRC	**0.612 ± 0.059**	0.593 ± 0.033	0.552 ± 0.024
ADENOMAS	**0.667 ± 0.058**	0.628 ± 0.054	0.604 ± 0.069
BIO_ML	**0.749 ± 0.024**	**0.749 ± 0.024**	0.744 ± 0.026
IBD_RELATIVES	**0.790 ± 0.016**	0.763 ± 0.052	0.767 ± 0.039
IBS	0.465 ± 0.051	0.462 ± 0.061	**0.494 ± 0.051**
CRC	0.555 ± 0.034	**0.561 ± 0.035**	0.521 ± 0.031
GC	**0.669 ± 0.063**	0.528 ± 0.052	0.596 ± 0.037
ESRD	**0.634 ± 0.036**	0.609 ± 0.037	0.586 ± 0.035
IBD	**0.857 ± 0.027**	0.821 ± 0.026	0.838 ± 0.024
IBDMDB	**0.674 ± 0.020**	0.521 ± 0.065	0.459 ± 0.074

The bolded values indicate the best results among the three expert allocation strategies

**Table 4:** Key Microorganism-Metabolite Associations Identified by ARD Mechanism

Microorganism	Metabolites
Bacteroides	mevalonate
Phocaeicola	7-ketodeoxycholate
Odoribacter	ursodeoxycholate
Alistipes	ursocholate
Parabacteroides	dihomo-linolenate
Agathobacter	hexadecadienoate
Blautia_A	isoursodeoxycholate
CAG—83	glycerophosphorylcholine
Faecalibacterium	hyocholate
Ruminiclostridium_E	12-dehydrocholate

**Table 5:** Microorganism-Metabolite Associations and Corresponding Metabolic Pathways

Microorganism	Metabolites	Metabolic pathway
Bacteroides, Odoribacter, Parabacteroides	Ursodeoxycholate, 7-Ketodeoxycholate	Bile acid metabolism [52, 53]
Bacteroides, Alistipes, Faecalibacterium	Dihomo-linolenate	Lipid metabolism [54–56]
Phocaeicola, Alistipes, Blautia_A	Glycerophosphorylcholine (GPC)	Phospholipid metabolism [54]

**Fig. 6:**
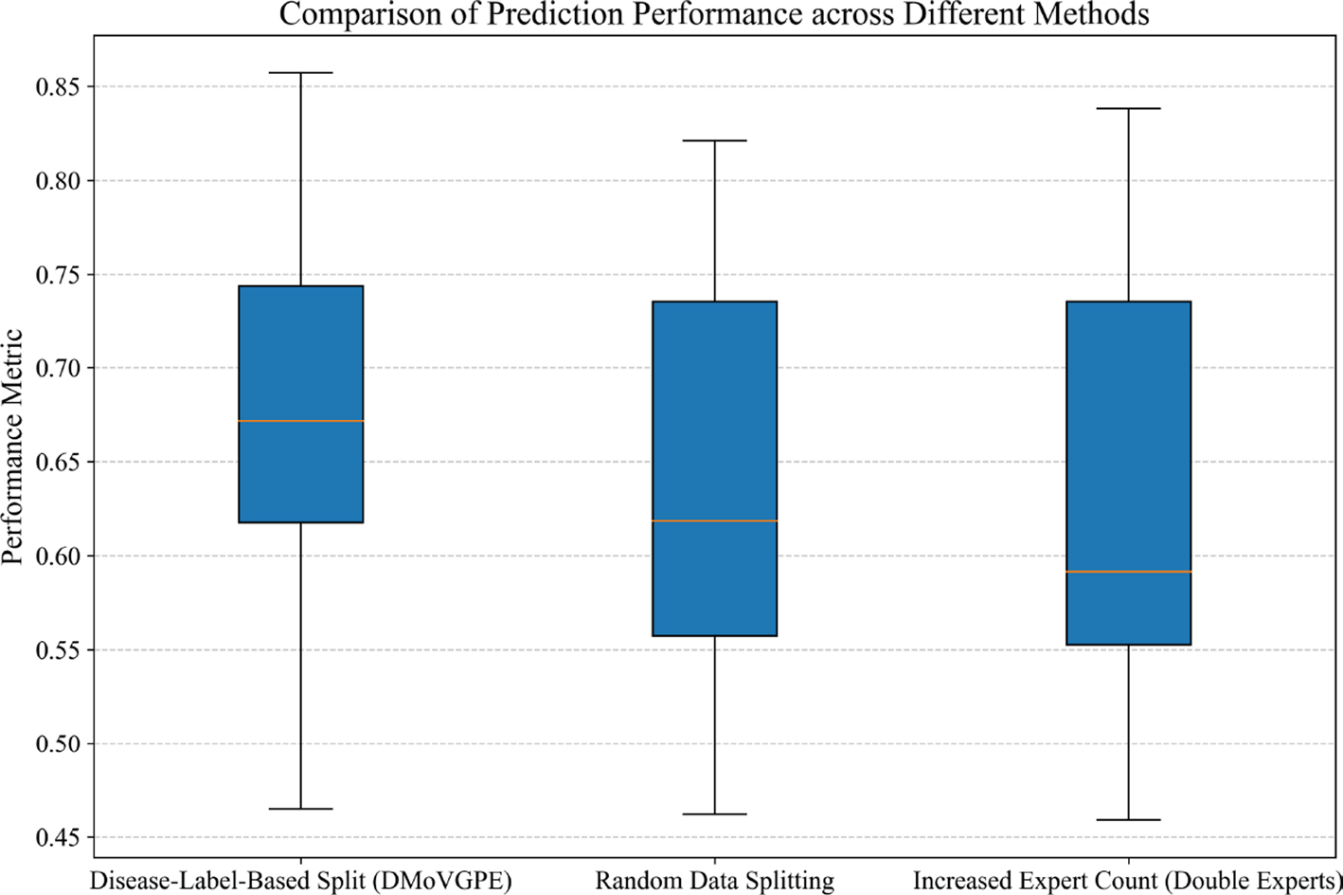
Box diagram of comparing analysis results

To facilitate a clearer comparison of these three strategies, a box plot is presented in Fig. 6. This visualization highlights the differences in performance across the methods. Specifically, the DMoVGPE method exhibits a higher median, indicating its superior overall performance. Moreover, the performance distribution of DMoVGPE is more concentrated, indicating lower variability and greater stability in its results. In contrast, the median performance of the random data splitting and increased expert count (double experts) methods is somewhat lower, with a broader distribution range, indicating more significant fluctuations in performance across certain datasets. These findings underscore the reliability and consistency of the DMoVGPE approach with phenotype-based split. This targeted allocation strategy improved prediction accuracy, especially in datasets with distinct health categories or limited sample sizes, demonstrating the practical advantages of DMoVGPE for metabolite prediction in clinical research.

## Leveraging automatic relevance determination for model interpretability

In this section, we utilized ARD to identify key features that significantly influence model predictions. By assigning inverse length scales to input dimensions, ARD automatically highlights features with greater predictive relevance, enabling effective feature selection and enhancing model interpretability.

**Fig. 7:**
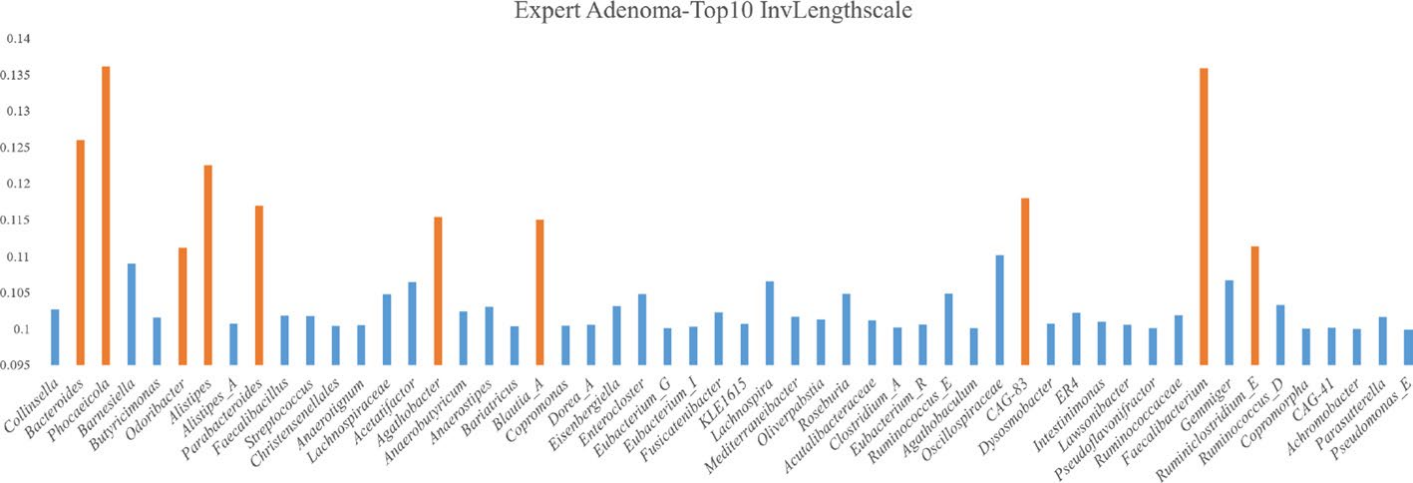
Inverse Lengthscales of the Adenoma Expert in the DMoVGPE Model

Using the ADENOMAS dataset as an example, as shown in Fig. 7, we selected the top ten microbial features based on the inverse length scale of the ADENOMAS expert and investigated their associations with adenoma through relevant literature. Tadashi et al. [43], explored the relationship between *Phocaeicola* and adenoma, revealing that *Phocaeicola* species, particularly *P. dorei*, are prevalent in both healthy individuals and adenoma patients. However, its levels decrease in cancerous tissues, indicating a potential protective role during early adenoma stages, which diminishes in advanced colorectal cancer (CRC). Jing et al. [44] further highlight *P. dorei* as one of the five most abundant bacteria in advanced adenomas, suggesting its role in altering the gut microbiome and increasing colorectal abnormalities. Vacante et al. [45] examined the association between *Faecalibacterium* and adenoma, noting a significant reduction in *Faecalibacterium prausnitzii* in adenoma patients. This reduction may correlate with adenoma development, compromising gut microbiome stability and increasing the risk of intestinal diseases. Similarly, Fu et al. [46] found that *Alistipes* abundance significantly increases in adenoma patients, suggesting a possible pathological role in adenoma development and inflammation. The research by Lin et al. [47] emphasized the potential importance of *Blautia_A* in adenoma, reporting significant changes in its abundance among adenoma patients. Certain strains of *Blautia* may influence gut health by affecting inflammatory responses and metabolic activities, thereby contributing to adenoma progression. Overall, these studies highlight the critical roles of specific gut microbiota in adenoma development and CRC risk.

**Fig. 8:**
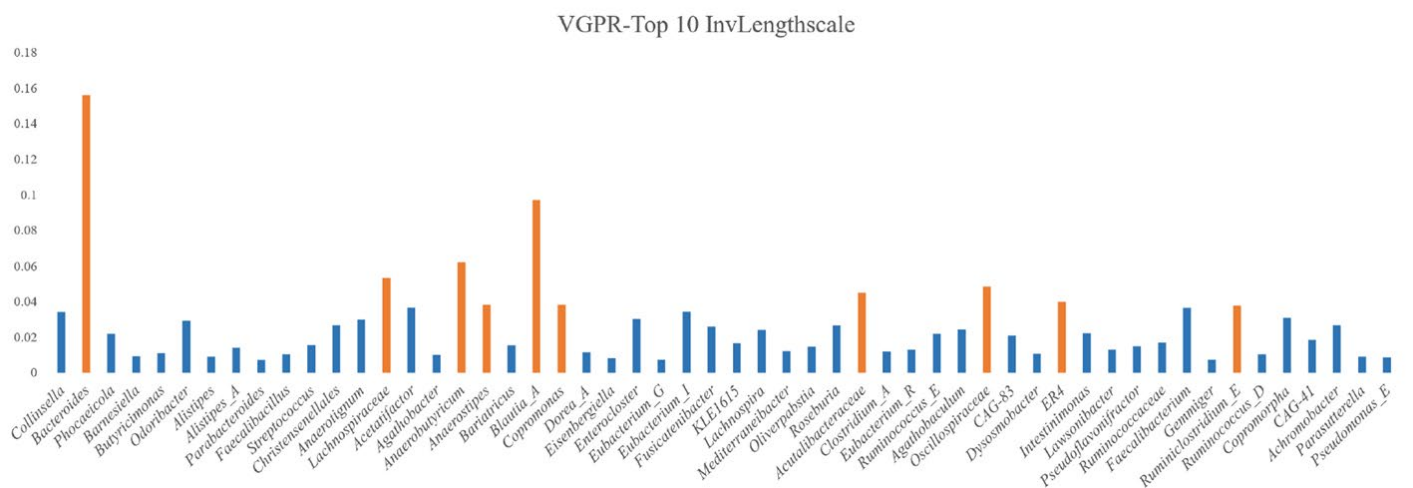
Inverse Lengthscales of the VGPR Model on the ADENOMAS Dataset

Figure 8 shows the ARD inverse length scales for VGPR on the ADENOMAS dataset. The results of the VGPR model's ARD suggest that its predictions are more focused on the overall characteristics of the adenoma patient population. In contrast, the DMoVGPE model's ARD is more geared toward identifying disease-specific features. Compared to DMoVGPE, it is evident that only Blautia_A appears among the top ten key microbes in VGPR. This not only highlights that DMoVGPE can identify more key microbes but also demonstrates its superior predictive performance. The broader selection of important microbes by DMoVGPE reflects its ability to capture more comprehensive features associated with the disease, leading to more accurate predictions.

**Fig. 9:**
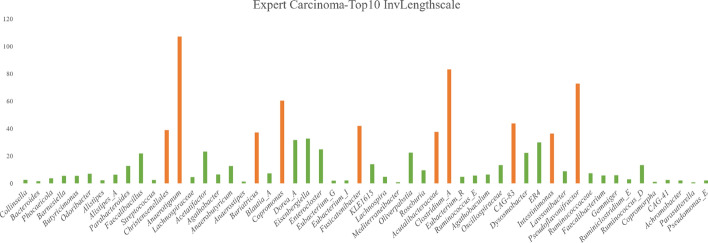
Inverse Lengthscales of the Carcinoma Expert in the DMoVGPE Model

Similarly, for the Carcinoma subset, the ARD mechanism in DMoVGPE becomes more focused on the Carcinoma disease state. As shown in Fig. 9, we identified the top ten microbial features based on the inverse length scale of the Carcinoma expert. In Xu et al.'s study [48], *Anaerotignum* was found to potentially influence gut microbiota, especially in patients experiencing ≥ grade 3 adverse events, which may link it to CRC. Czepiel et al. [49] reported a significant increase in *Clostridium_A* abundance among adenoma patients, suggesting its involvement in adenoma development and progression through modulation of gut inflammation and metabolism. Li et al. [49] identified *Pseudoflavonifractor* as enriched in adenoma patients, indicating its role in altering microbial balance and increasing CRC risk. Ruiz-Saavedra et al. [50] noted a significant decrease in *Christensenellales* abundance in adenoma patients, suggesting its protective role against tumor development. Finally, Tran N.T et al. [51] highlighted the decreased abundance of *Fusicatenibacter* in adenoma patients, which may indicate its potential to protect against adenomas and CRC. These findings highlight the effectiveness of incorporating ARD into the DMoVGPE model. By accurately identifying key microbial features—defined as taxa that significantly contribute to metabolite prediction through their weights in the model—this approach enhances the model's predictive accuracy and interpretability in complex, high-dimensional microbiome datasets. Beyond improving feature selection and prediction, it provides deeper insights into the intricate relationships between the gut microbiome and disease progression. These advances not only propel microbiome research forward but also pave the way for targeted therapeutic strategies and precision medicine in microbiome-related diseases.

Below, we present the key microorganisms and their associated metabolites:

Additionally, we have included an analysis of the metabolic pathways associated with these microorganisms and metabolites:

The application of ARD not only identified key microbial features associated with adenoma development but also revealed their functional roles in critical metabolic pathways, providing deeper insights into the interplay between gut microbiota and host metabolism. For instance, *Bacteroides*, *Odoribacter*, and *Parabacteroides* were linked to bile acid metabolism through their production of ursodeoxycholate and 7-ketodeoxycholate. These secondary bile acids, generated via microbial transformation of primary bile acids, regulate lipid absorption and inflammatory signaling by modulating nuclear receptors such as FXR and TGR5. Dysregulation of bile acid metabolism has been implicated in colorectal carcinogenesis, as excessive 7-ketodeoxycholate may promote oxidative stress and epithelial damage, while reduced levels of anti-inflammatory ursodeoxycholate could compromise gut barrier integrity [52, 53].

Similarly, *Bacteroides*, *Alistipes*, and *Faecalibacterium* were associated with lipid metabolism, particularly the synthesis of dihomo-linolenate (20:3n6), an ω-6 polyunsaturated fatty acid precursor to pro-inflammatory eicosanoids. Elevated dihomo-linolenate levels may reflect a pro-tumorigenic microenvironment characterized by enhanced cyclooxygenase-2 (COX-2) activity and prostaglandin E2 (PGE2) production, both known drivers of adenoma progression [54–56]. Conversely, *Faecalibacterium*, a prominent butyrate producer, typically exerts anti-inflammatory effects; its decline in adenoma patients suggests a loss of protective mechanisms that normally counteract lipid-mediated inflammation. Phospholipid metabolism emerged as another critical pathway, mediated by *Phocaeicola*, *Alistipes*, and *Blautia_A*, which generate glycerophosphorylcholine (GPC). GPC serves as a key intermediate in membrane phospholipid remodeling and choline metabolism, supporting epithelial cell repair and signal transduction. Reduced GPC levels may impair mucosal regeneration, exacerbating inflammation-driven DNA damage in the colorectal epithelium [54]. Notably, *Blautia_A*’s association with isoursodeoxycholate further highlights its dual role in bile acid modification and short-chain fatty acid production, linking lipid and phospholipid pathways to gut homeostasis. The interconnectedness of these pathways underscores the systemic impact of microbiota-driven metabolism on adenoma pathogenesis. For example, CAG-83 and Ruminiclostridium_E contribute to glycerophosphorylcholine and 12-dehydrocholate production, respectively, bridging phospholipid and bile acid metabolism. These interactions may influence cellular energy metabolism, oxidative stress, and immune responses, collectively shaping the metabolic landscape of early adenoma lesions. The observed increase in Alistipes-derived ursocholate and Agathobacter-associated hexadecadienoate further suggests microbial adaptation to a dysbiotic environment, marked by altered lipid peroxidation and compromised detoxification mechanisms.

In summary, the ARD mechanism has proven to be a valuable approach for identifying key microbial features and their functional roles in critical metabolic pathways associated with disease development. The interactions between gut microbiota and host metabolism, particularly through bile acid, lipid, and phospholipid metabolism, reveal complex mechanisms that could influence disease progression. The identified microbial metabolites serve not only as potential biomarkers for disease but also as targets for therapeutic interventions aimed at restoring microbial balance and metabolic homeostasis. These findings highlight the systemic influence of the microbiome on disease states and provide a basis for future research focused on microbiota-based strategies for improving health outcomes and preventing disease progression.

## Conclusion

In this work, a novel computational framework called DMoVGPE is presented for predicting metabolite profiles from microbiome data. Our experimental results demonstrate the model’s superiority across multiple datasets, consistently outperforming traditional and modern approaches in metabolite prediction. The evaluation of expert allocation strategies highlighted the advantages of partitioning datasets based on phenotype labels, allowing each expert to specialize in these subsets and significantly enhancing predictive performance. Furthermore, the integration of expert level automatic relevance determination enabled the identification of key microbial features influencing metabolite production, offering valuable insights into the complex relationships between the gut microbiome and host metabolism. These findings not only deepen our understanding of microbiome-host interactions but also hold significant implications for personalized medicine and disease treatment strategies, paving the way for more targeted and effective therapeutic interventions. While the DMoVGPE model has demonstrated robust performance, there remain opportunities for further refinement to address specific challenges. In its current implementation, the model employs the same kernel function for all expert models, which may not fully capture the diverse patterns across different phenotypes. This uniformity could limit the model’s ability to adapt to the unique characteristics of each phenotype, particularly in highly heterogeneous datasets. Additionally, while the model demonstrates strong performance across multiple datasets, its scalability and efficiency could be further enhanced to handle larger datasets and a broader range of health conditions. To address these limitations, future research could explore several promising directions. One approach is to develop adaptive kernel functions tailored to specific phenotypes, allowing the model to better capture the distinct patterns inherent in each subset of data. Furthermore, integrating attention mechanisms into the gating network could improve its ability to capture complex relationships within the data, enabling more nuanced and accurate predictions. These advancements would not only refine the model’s predictive performance but also expand its applicability to a wider range of clinical and biological challenges.

## Supplementary Information

Supplementary Material 1.

## Data Availability

All data files can be found in the pertaining github repository under the https://github.com/borenstein-lab/microbiome-metabolome-curated-data/tree/main/data/processed_data folder. All data processing, analysis, calculation, and visualization are Python programs, and all workflows are described in the Methods section. All code and data for running DMoVGPE can be found at https://github.com/qinghuiwww/DMoVGPE/tree/master.
