# Supplementary material for: DMoVGPE: predicting gut microbial associated metabolites profiles with deep mixture of variational Gaussian Process experts
Source: BMC Bioinformatics. 2025 Mar 27;26:93. doi: 10.1186/s12859-025-06110-7 (PMC11951675; doi:10.1186/s12859-025-06110-7)
Supplement: Supplementary file 1 — Supplementary Material 1. [file 12859_2025_6110_MOESM1_ESM.docx]

Appendix

## Detailed Molecular Mechanisms of Key Metabolites (TMAO, SCFAs) and Their Impact on Health

Trimethylamine-N-oxide (TMAO) is a metabolite produced by gut microbiota through the fermentation of dietary compounds such as choline, phosphatidylcholine, and L-carnitine, which are found predominantly in red meat. TMAO has been implicated in several adverse health outcomes, particularly in cardiovascular disease. The molecular mechanisms by which TMAO influences cardiovascular health include its ability to promote atherosclerosis through the enhancement of cholesterol deposition in arterial walls, reduction of reverse cholesterol transport, and stimulation of endothelial cell inflammation. These processes collectively contribute to plaque formation and the development of arterial stiffness. Furthermore, TMAO plays a role in immune modulation, as it has been shown to influence the function of macrophages and monocytes, leading to an increase in systemic inflammation, which is closely associated with the pathogenesis of metabolic and cardiovascular diseases [1].

Short-chain fatty acids (SCFAs), including acetate, propionate, and butyrate, are products of gut microbial fermentation of dietary fibers. SCFAs exert multiple physiological effects that contribute to health. Butyrate, in particular, is a critical energy source for colonic epithelial cells, and its presence strengthens the gut barrier by promoting the expression of tight junction proteins. This enhancement of intestinal barrier integrity prevents gut leakage, which can lead to systemic inflammation and is linked to various chronic diseases. SCFAs also have significant effects on immune function, where they modulate immune responses by activating GPR41 and GPR43 receptors on immune cells. These actions promote the expansion of regulatory T cells (Tregs), which help to control excessive immune responses and maintain immune tolerance, thus playing a protective role in autoimmune and inflammatory diseases. Additionally, SCFAs regulate metabolic processes, influencing lipid metabolism, glucose metabolism, and insulin sensitivity. They affect adiposity and obesity by modulating hepatic and adipose tissue metabolism, and also influence the secretion of gut hormones that are involved in appetite regulation. SCFAs are further recognized for their role in modulating neuroinflammation, with evidence suggesting that they can cross the blood-brain barrier and influence central nervous system conditions such as anxiety, depression, and neurodegenerative diseases [2] [3].

Considering these insights, understanding the specific molecular mechanisms through which metabolites like TMAO and SCFAs influence human health underscores the necessity of accurately predicting metabolite profiles. Given the complex and dynamic nature of microbiome-metabolite interactions, direct measurement of metabolites through metabolomics is often costly and time-consuming. As a result, leveraging microbial composition data to predict metabolites offers a more accessible and cost-effective alternative. By utilizing the microbiome to predict metabolites, we can gain a deeper understanding of the biochemical processes governing health and disease, providing valuable insights for personalized healthcare strategies, disease prevention, and the development of novel therapeutic approaches [4] [5] [6].

## Dataset Collection, Processing, and Quality Control

This section provides detailed information about the collection, processing, and quality control steps applied to the 14 publicly available human gut microbial metabolite datasets used in this study. The datasets were sourced from the curated repository maintained by the Borenstein Lab, accessible at: https://github.com/borenstein-lab/microbiome-metabolome-curated-data. Additional details about the datasets can be found on the Data Overview Wiki.

The datasets were collected from diverse cohorts, encompassing individuals with varying health conditions, dietary habits, and geographic backgrounds. These cohorts include individuals with inflammatory bowel disease (IBD), colorectal cancer (CRC), gastric cancer (GC), irritable bowel syndrome (IBS), autism spectrum disorder (ASD), type 1 diabetes (INFANTS-DIABETES), late-onset sepsis (PRETERMS) and end-stage renal disease (ESRD), as well as healthy controls (e.g., BIO_ML). The geographic diversity of the cohorts ensures a broad representation of human gut microbial and metabolic profiles, enhancing the generalizability of our findings.

To ensure the quality and consistency of the data, several preprocessing steps were applied. Microbial and metabolite features with more than 50% of their abundance values equal to zero were removed to reduce data sparsity and dimensionality, minimizing the risk of overfitting and improving the reliability of downstream analyses. Zero abundances were replaced with very small values (pseudo-counts) to address numerical issues that arise during logarithmic transformations, a critical step for handling compositional data. The data were normalized using the central logarithmic ratio (clr) transformation.

Rigorous quality control measures were applied at each stage of data collection and processing. Standardized protocols were followed for sample collection, DNA extraction, and metabolite profiling to ensure consistency and reproducibility across studies. For metagenomic data, quality control steps included trimming low-quality reads, removing adapter sequences, and filtering chimeric sequences. For metabolomic data, quality control involved peak alignment, noise reduction, and removal of low-quality spectra. Metadata tables were carefully curated to include detailed information about sample sources, health status, and experimental conditions, ensuring accurate interpretation of the data and facilitating integrative analyses.

The table below summarizes the key characteristics of each dataset, including sample size, number of subjects, metagenomics and metabolomics approaches. For more detailed information, please refer to the Data Overview Wiki.

Table 1. Description of Datasets and Associated Processing Methods

| Dataset | Cohort description | No. samples | No. subjects | Metagenomics approach | Metabolomics approach |
| --- | --- | --- | --- | --- | --- |
| MFGM [7] | Infants on different diets during their 1st year of life | 277 | 80 | 16S V4 | Targeted, 1H-NMR |
| ASD [8] | Children with autism and neurotypical children | 44 [21] | 44 [21] | 16S V2-V3 | Targeted, 1H-NMR |
| INFANTS_DIABETES [9] | Longitudinal samples from children at risk for T1D (DIABIMMUNE cohort) | 103 [37] | 19 [8] | 16S V4 | Untargeted, Four complimentary LC-MS methods |
| PRETERMS [10] | Preterm infants during their first 6 months of life. Some developed LOS/NEC | 75 [37] | 32 [21] | 16S V3-V4 | Untargeted, GC-MS |
| SINHA_CRC [11] | CRC patients and controls | 131 [89] | 131 [89] | 16S V3-V4 | Untargeted, HPLC-GC/MS-MS |
| ADENOMAS [12] | Patients with advanced colorectal adenomas, CRC, and controls | 240 [102] | 240 [102] | 16S V3-V5 | Untargeted, UPLC-MS/MS |
| BIO_ML [13] | Longitudinal samples from healthy BIO-ML (stool bank) donors | 164 | 83 | WGSS + 16S V4 | Untargeted, Four complimentary LC-MS methods |
| IBD_RELATIVES [14] | IBD patients and their first degree (healthy) relatives | 90 [54] | 90 [54] | 16S V4 | Untargeted, UPLC/ToFMS |
| IBS [15] | Longitudinal samples from patients with IBS and controls | 444 [139] | 75 [24] | Shotgun | Targeted, 1H-NMR + LC-MS/MS |
| CRC [16] | Patients with colonoscopy findings from normal to stage 4 CRC | 347 [127] | 347 [127] | Shotgun | Targeted, CE-TOFMS |
| GC [17] | Patients with a history of gastrectomy for GC | 96 [54] | 96 [54] | Shotgun | Targeted, CE-TOFMS |
| ESRD [18] | Adults with ESRD and controls | 287 [67] | 287 [67] | Shotgun | Untargeted, GC-MS |
| IBD [19] | IBD patients and controls (PRISM cohort + A validation cohort) | 220 [56] | 220 [56] | Shotgun | Untargeted, Four complimentary LC-MS methods |
| IBDMDB [20] | HMP2 cohort: Longitudinal samples from IBD patients and controls | 382 [104] | 105 [26] | Shotgun | Untargeted, Four complimentary LC-MS methods |

The number of samples/subjects from the ‘control’ study group is noted in square brackets (e.g., 44 [21] indicates 44 samples, including 21 controls).

## Performance comparison between DMoVGPE and existing methods

To provide a more comprehensive evaluation of the DMoVGPE model in metabolite prediction, we compared it against several machine learning methods, categorized into four main groups: (1) Linear models: including Linear Regression (LR), L1 regularization(Lasso), MelonnPan, and Partial Least Squares (PLS); (2) Nonlinear models: such as Support Vector Machine (SVM) and Relevance Vector Machine (RVM); (3) Ensemble tree models: comprising Random Forest (RF), XGBoost (XGB) and LightGBM (LGB); and (4) Deep neural network models: including SparseNED, MiMeNet, and mNODE. All models were trained on the same preprocessed data. MelonnPan, SparseNED, MiMeNet, and mNODE were run using their default settings from publicly available GitHub repositories, while the remaining models were implemented in scikit-learn, with optimal hyperparameters determined via grid search.

We utilized Spearman's rank correlation coefficient (SCC) to evaluate the agreement between predicted and observed metabolite abundances. For each sample, we calculated the correlation between the predicted and true metabolite abundances. The average SCC across all samples provides a measure of the overall prediction accuracy of the model. We focus on the top ten ranked metabolites to assess the model's performance. Furthermore, a 5-fold cross-validation was applied to all models to ensure robust and unbiased performance evaluation across different data splits, resulting in the reported outcomes being presented as average values $\pm$ standard deviation in Table 1. The best method for predicting metabolites in each dataset is highlighted in bold. while the second-best results are underlined. Furthermore, due to the high metabolite dimensionality of the IBDMDB dataset, models like SVM, RF and XGB require substantial computational resources for metabolite predictions, leading to dimensionality explosion when processing the feature matrices; hence, we marked their results as ‘/’.

| Table 2. Results of Benchmarking Models | Dataset | MFGM | ASD | INFANTS_DIABETES | PRETERMS | SINHA_CRC | ADENOMAS | BIO_ML | IBD_RELATIVES | IBS | CRC | GC | ESRD | IBD | IBDMDB | Average |
| --- | --- | --- | --- | --- | --- | --- | --- | --- | --- | --- | --- | --- | --- | --- | --- | --- |
|  | LR | 0.4852±0.0364 | 0.5437±0.1136 | 0.6434±0.0888 | 0.7561±0.0426 | 0.5507±0.0372 | 0.5780±0.0387 | 0.6417±0.0251 | 0.6402±0.0396 | 0.1869±0.0494 | 0.4778±0.0203 | 0.6463±0.0431 | 0.6284±0.0261 | 0.8529±0.0191 | 0.6408±0.0212 | 0.5909 |
|  | Lasso | 0.5026±0.0399 | 0.5927±0.1051 | 0.6620±0.0133 | 0.7791±0.0489 | 0.5463±0.0549 | 0.5930±0.0459 | 0.6947±0.0384 | 0.6676±0.0352 | 0.3038±0.0467 | 0.5003±0.0197 | 0.7021±0.0708 | 0.6024±0.0260 | 0.8419±0.0201 | 0.6332±0.0223 | 0.6158 |
|  | MelonnPan | 0.5457±0.0312 | 0.5833±0.0287 | 0.6796±0.0558 | 0.7146±0.0151 | 0.5090±0.0273 | 0.6539±0.0419 | 0.7114±0.0586 | 0.6300±0.0274 | 0.2476±0.0337 | 0.4860±0.0355 | 0.3123±0.0461 | 0.4188±0.0459 | 0.7077±0.0228 | 0.6351±0.0399 | 0.5596 |
|  | PLS | 0.5067±0.0334 | 0.5509±0.1388 | 0.6928±0.0629 | 0.7676±0.0451 | 0.5465±0.0735 | 0.6198±0.0423 | 0.6845±0.0310 | 0.7489±0.0378 | 0.2309±0.0252 | 0.5501±0.0217 | 0.6593±0.0438 | 0.6130±0.0211 | 0.8515±0.0218 | 0.6606±0.0306 | 0.6202 |
|  | SVM | 0.4867±0.0306 | 0.5680±0.0964 | 0.6724±0.0638 | 0.7348±0.0401 | 0.5390±0.0479 | 0.5732±0.0291 | 0.6499±0.0202 | 0.6596±0.0355 | 0.2022±0.0433 | 0.4856±0.0229 | 0.6572±0.0460 | 0.6316±0.0232 | 0.8520±0.0212 | / | 0.5932 |
|  | RVM | 0.4995±0.0432 | 0.5604±0.0998 | 0.7025±0.0359 | 0.7756±0.0332 | 0.5607±0.0574 | 0.6153±0.0417 | 0.7048±0.0311 | 0.7121±0.0202 | 0.1992±0.0427 | 0.4804±0.0211 | 0.6529±0.0569 | 0.6289±0.0234 | 0.8536±0.0208 | / | 0.6112 |
|  | RF | 0.4954±0.0434 | 0.5531±0.0924 | 0.7149±0.0233 | 0.7784±0.0314 | 0.5618±0.0466 | 0.6213±0.0550 | 0.7145±0.0386 | 0.7583±0.0529 | 0.2279±0.0502 | 0.5568±0.0249 | 0.7706±0.0515 | 0.6270±0.0190 | 0.8472±0.0218 | 0.6590 ± 0.0237 | 0.6347 |
|  | XGB | 0.4540±0.0281 | 0.4649±0.0585 | 0.6573±0.0152 | 0.7645±0.0383 | 0.5482±0.0360 | 0.5832±0.0325 | 0.6986±0.0209 | 0.7151±0.0494 | 0.2476±0.0272 | 0.5064±0.0150 | 0.7442±0.0424 | 0.5832±0.0153 | 0.8339±0.0180 | / | 0.6001 |
|  | LGB | 0.4475±0.0218 | 0.422±0.0321 | 0.6858±0.0295 | 0.7696±0.0433 | 0.5539±0.0380 | 0.5981±0.0339 | 0.7007±0.0265 | 0.7285±0.0445 | 0.2559±0.0285 | 0.5246±0.0107 | 0.7208±0.0362 | 0.6217±0.0162 | 0.8437±0.0218 | / | 0.6056 |
|  | SparseNED | 0.4901±0.0398 | 0.5203±0.0817 | 0.7135±0.0158 | 0.7431±0.0374 | 0.5624±0.0282 | 0.5307±0.0387 | 0.6863±0.0329 | 0.7564±0.0459 | 0.2654±0.0217 | 0.4997±0.0154 | 0.6065±0.0352 | 0.5990±0.0166 | 0.8465±0.0258 | 0.6633±0.0216 | 0.6059 |
|  | MiMeNet | 0.5463±0.0052 | 0.4059±0.0282 | 0.5770±0.0208 | 0.6853±0.0164 | 0.3956±0.0165 | 0.5416±0.0054 | 0.7351±0.0033 | 0.6489±0.0082 | 0.3690±0.0056 | 0.5200±0.0068 | 0.6153±0.0158 | 0.4981±0.0111 | 0.7027±0.0155 | 0.5509±0.0028 | 0.5565 |
|  | mNODE | 0.5613±0.0953 | 0.6957±0.0723 | 0.6824±0.0384 | 0.7399±0.1060 | 0.5238±0.0370 | 0.6241±0.0531 | 0.6666±0.0892 | 0.7034±0.0547 | 0.4410±0.0873 | 0.4076±0.0835 | 0.6330±0.0674 | 0.5649±0.0418 | 0.8274±0.0357 | 0.5510±0.0461 | 0.6159 |
|  | VGPR | 0.5252±0.0425 | 0.6203±0.0801 | 0.7203±0.0474 | 0.7853±0.0397 | 0.5808±0.0352 | 0.6366±0.0429 | 0.7223±0.0265 | 0.7789±0.0570 | 0.3054±0.0531 | **0.5749±0.0172** | **0.7723±0.0425** | **0.6446±0.0186** | 0.8567±0.0252 | 0.6664±0.0173 | 0.6554 |
|  | DMoVGPE | **0.5659±0.0420** | **0.7032±0.1042** | **0.7280±0.0437** | **0.7966±0.0333** | **0.6121±0.0588** | **0.6671±0.0579** | **0.7488±0.0242** | **0.7904±0.0163** | **0.4645±0.0509** | 0.5548±0.0344 | 0.6694±0.0632 | 0.6341±0.0361 | **0.8574±0.0269** | **0.6737 ± 0.0196** | **0.6762** |

The results summarized in Table 1 indicate that the DMoVGPE model consistently outperforms various traditional and modern predictive models across multiple datasets, ranking first in 11 datasets. For the CRC and ESRD datasets, it achieves the second-best performance. For instance, in the ADENOMAS dataset, DMoVGPE achieved SCC of 0.6671±0.0579, surpassing linear models such as Linear Regression and Lasso, which often encounter challenges when dealing with nonlinear relationships in the data and may overlook complex interactions. Similarly, while ensemble models like Random Forest and XGBoost demonstrate robust performance, they are often constrained by their inability to effectively model the inherent uncertainty and smoothness in the relationships between microbiome and metabolome data. This limitation leads them to poorer performance on datasets like IBD, where DMoVGPE demonstrates a clear advantage. These results highlight not only the effectiveness of DMoVGPE in capturing the complex relationships inherent in microbiome and metabolome data but also its ability to generalize across diverse disease states.

Table 3. All metabolites predict SCC mean results

| Dataset | LR | Lasso | PLS | SVM | RVM | RF | XGB | LGB | SparseNED | MiMeNet | mNODE | VGPR | DMoVGPE |
| --- | --- | --- | --- | --- | --- | --- | --- | --- | --- | --- | --- | --- | --- |
| MFGM | 0.201 | 0.205 | 0.208 | 0.196 | 0.213 | 0.225 | 0.189 | 0.196 | 0.197 | 0.296 | 0.240 | 0.255 | **0.327** |
| ASD | 0.031 | 0.098 | 0.004 | 0.056 | 0.049 | 0.048 | 0.009 | 0.126 | 0.049 | 0.224 | 0.040 | 0.239 | **0.270** |
| INFANTS_DIABETES | 0.372 | 0.335 | **0.433** | 0.198 | 0.291 | 0.279 | 0.264 | 0.327 | 0.285 | 0.231 | 0.146 | 0.328 | 0.353 |
| PRETERMS | 0.183 | 0.231 | 0.235 | 0.147 | 0.188 | 0.248 | 0.172 | 0.159 | 0.195 | 0.287 | 0.212 | 0.251 | **0.303** |
| SINHA_CRC | 0.086 | 0.069 | 0.074 | 0.118 | 0.146 | 0.114 | 0.100 | 0.058 | 0.064 | 0.115 | 0.098 | 0.230 | **0.251** |
| ADENOMAS | 0.171 | 0.222 | 0.227 | 0.228 | 0.261 | 0.246 | 0.190 | 0.229 | 0.156 | 0.238 | 0.298 | 0.260 | **0.319** |
| BIO_ML | 0.222 | 0.302 | 0.262 | 0.276 | 0.340 | 0.287 | 0.331 | 0.284 | 0.288 | 0.331 | 0.228 | 0.304 | **0.345** |
| IBD_RELATIVES | 0.040 | 0.044 | 0.037 | 0.031 | 0.063 | 0.094 | 0.057 | 0.081 | 0.078 | 0.077 | 0.066 | 0.094 | **0.251** |
| IBS | 0.104 | 0.232 | 0.119 | 0.113 | 0.115 | 0.119 | 0.061 | 0.125 | 0.022 | 0.205 | 0.271 | 0.243 | **0.306** |
| CRC | 0.218 | 0.198 | 0.283 | 0.210 | 0.209 | 0.256 | 0.193 | 0.227 | 0.210 | 0.271 | 0.151 | 0.272 | **0.289** |
| GC | 0.230 | 0.244 | 0.272 | 0.171 | 0.144 | **0.392** | 0.300 | 0.293 | 0.187 | 0.251 | 0.054 | 0.365 | 0.237 |
| ESRD | 0.234 | 0.192 | 0.252 | 0.237 | 0.234 | 0.235 | 0.190 | 0.195 | 0.207 | 0.291 | **0.339** | 0.234 | 0.258 |
| IBD | 0.352 | 0.300 | 0.378 | 0.364 | 0.366 | 0.357 | 0.317 | 0.329 | 0.360 | 0.284 | 0.236 | 0.324 | **0.389** |
| IBDMDB | 0.163 | 0.134 | 0.169 | \ | \ | **0.201** | \ | \ | 0.186 | 0.196 | 0.011 | 0.177 | 0.200 |

Table 4. Prediction of SCC mean value of the top 50 metabolites

| Dataset | LR | Lasso | PLS | SVM | RVM | RF | XGB | LGB | SparseNED | MiMeNet | mNODE | VGPR | DMoVGPE |
| --- | --- | --- | --- | --- | --- | --- | --- | --- | --- | --- | --- | --- | --- |
| MFGM | 0.298 | 0.308 | 0.302 | 0.301 | 0.323 | 0.302 | 0.307 | 0.297 | 0.310 | 0.395 | 0.405 | 0.356 | **0.415** |
| ASD | 0.061 | 0.126 | 0.037 | 0.084 | 0.076 | 0.068 | 0.035 | 0.167 | 0.090 | 0.242 | 0.148 | 0.311 | **0.366** |
| INFANTS_DIABETES | 0.598 | 0.566 | **0.723** | 0.478 | 0.625 | 0.493 | 0.547 | 0.573 | 0.568 | 0.446 | 0.419 | 0.589 | 0.606 |
| PRETERMS | 0.578 | 0.637 | 0.621 | 0.589 | 0.639 | 0.540 | 0.603 | 0.587 | 0.580 | 0.566 | 0.630 | 0.639 | **0.667** |
| SINHA_CRC | 0.431 | 0.436 | 0.431 | 0.441 | 0.529 | 0.313 | 0.450 | 0.448 | 0.417 | 0.329 | 0.425 | 0.388 | **0.561** |
| ADENOMAS | 0.457 | 0.525 | 0.553 | 0.529 | 0.581 | 0.492 | 0.499 | 0.550 | 0.438 | 0.477 | 0.590 | 0.575 | **0.623** |
| BIO_ML | 0.527 | 0.618 | 0.560 | 0.569 | 0.645 | 0.568 | **0.652** | 0.578 | 0.595 | 0.650 | 0.608 | 0.643 | 0.651 |
| IBD_RELATIVES | 0.559 | 0.573 | 0.661 | 0.525 | 0.583 | 0.534 | 0.592 | 0.633 | 0.674 | 0.544 | 0.661 | 0.659 | **0.729** |
| IBS | 0.104 | 0.232 | 0.119 | 0.113 | 0.115 | 0.119 | 0.061 | 0.125 | 0.022 | 0.205 | 0.271 | 0.306 | **0.343** |
| CRC | 0.416 | 0.389 | 0.470 | 0.395 | 0.395 | 0.429 | 0.378 | 0.427 | 0.403 | 0.453 | 0.424 | 0.399 | **0.487** |
| GC | 0.454 | 0.583 | 0.502 | 0.429 | 0.395 | 0.643 | 0.629 | 0.557 | 0.426 | 0.440 | 0.348 | **0.647** | 0.417 |
| ESRD | 0.491 | 0.487 | **0.537** | 0.523 | 0.519 | 0.485 | 0.472 | 0.492 | 0.485 | 0.452 | 0.533 | 0.468 | 0.517 |
| IBD | 0.828 | 0.815 | **0.837** | 0.801 | 0.801 | 0.760 | 0.791 | 0.834 | 0.809 | 0.679 | 0.700 | 0.826 | 0.828 |
| IBDMDB | 0.580 | 0.550 | 0.579 | \ | \ | 0.554 | \ | \ | 0.601 | 0.525 | 0.458 | 0.593 | **0.605** |

Table 5. Number of metabolites with SCC > 0.5

| Dataset | LR | Lasso | PLS | SVM | RVM | RF | XGB | LGB | SparseNED | MiMeNet | mNODE | VGPR | DMoVGPE |
| --- | --- | --- | --- | --- | --- | --- | --- | --- | --- | --- | --- | --- | --- |
| MFGM | 4 | 5 | 6 | 4 | 7 | 1 | 4 | 0 | 2 | 10 | 9 | 5 | **14** |
| ASD | 4 | 8 | 5 | 6 | 6 | 0 | 4 | 6 | 8 | 0 | 3 | 4 | **16** |
| INFANTS_DIABETES | 72 | 46 | **87** | 19 | 48 | 18 | 30 | 47 | 39 | 10 | 6 | 44 | 48 |
| PRETERMS | 45 | **74** | 57 | 48 | 73 | 31 | 49 | 50 | 46 | 43 | 61 | 68 | **74** |
| SINHA_CRC | 9 | 11 | 6 | 7 | 29 | 0 | 14 | 11 | 9 | 0 | 10 | 30 | **46** |
| ADENOMAS | 12 | 28 | 33 | 38 | 59 | 19 | 17 | 40 | 6 | 10 | 69 | 38 | **72** |
| BIO_ML | 27 | 77 | 35 | 50 | **112** | 50 | 95 | 50 | 62 | 80 | 72 | 104 | 106 |
| IBD_RELATIVES | 36 | 43 | 67 | 28 | 47 | 28 | 53 | 63 | 73 | 35 | **150** | 111 | 127 |
| IBS | 0 | 0 | 0 | 0 | 0 | 0 | 0 | 0 | 0 | 0 | **3** | 0 | **3** |
| CRC | 6 | 6 | 11 | 2 | 2 | 7 | 4 | 5 | 5 | 13 | 9 | 5 | **20** |
| GC | 13 | 31 | 19 | 11 | 8 | **62** | 46 | 36 | 14 | 13 | 22 | 38 | 42 |
| ESRD | 21 | 22 | 33 | 26 | 26 | 19 | 16 | 23 | 20 | 3 | **44** | 16 | 28 |
| IBD | 2307 | 1683 | 2633 | 2205 | 2237 | 2155 | 1637 | 2119 | 2300 | 1051 | 1089 | 2574 | **2718** |
| IBDMDB | 386 | 94 | 295 | \ | \ | 314 | \ | \ | 512 | 95 | 4 | 372 | **773** |

MelonnPan introduces the concept of "well-predicted metabolites," a criterion also adopted by mNODE, which designates metabolites as "well-predicted" only if their SCC exceeds a predefined threshold. Similarly, based on the results in Tables 3, 4, and 5 (comparing model performance using three metrics: the average SCC of all predicted metabolites, the average SCC of the top 50 predicted metabolites, and the number of metabolites with SCC greater than 0.5, respectively, with the highest-ranking results highlighted in **bold**), we evaluated the performance of the DMoVGPE model against other methods across 14 datasets. Among these diverse approaches, our DMoVGPE model consistently demonstrated outstanding performance. The results indicate that the DMoVGPE model ranked first in approximately 10 out of these 14 datasets, showcasing a significant advantage over other models. This finding further underscores the strengths of the DMoVGPE model, particularly in capturing disease-specific features and achieving more accurate metabolite predictions through its mixture of experts framework.

**Computational Cost Comparisons**

In this section, we provide detailed computational cost comparisons between DMoVGPE and other baseline methods, including VGPR, MiMeNet, mNODE, MelonnPan, and RVM. The training times are reported for each dataset, along with the hardware configurations used for the experiments. All experiments were conducted on the following hardware configurations:

Server: CPU: Intel(R) Xeon(R) Silver 4210 @ 2.20GHz; RAM: 128 GB.

Local Machine: CPU: Intel(R) Core(TM) i5-8300H @ 2.30GHz; RAM: 16 GB.

**Notes on Experimental Setup**

**mNODE**: The mNODE experiments were run locally using a Julia implementation. The training times reflect the performance on the local machine.

**MelonnPan**: The MelonnPan experiments were also run locally using an R implementation. Due to shared server usage, the CPU resources were occasionally fully utilized, which may have introduced minor variations in the reported training times. However, these variations do not affect the overall comparison or conclusions.

The table below summarizes the training times for DMoVGPE and the baseline methods across all datasets. The times are reported in seconds (s), minutes (m), hours (h), or days (d), depending on the magnitude.

Table 6 Comparison of Computational Efficiency Across Methods on Various Datasets

| Dataset | LR | Lasso | MelonnPan | PLS | SVM | RVM | RF | XGB | LGB | SparseNED | MiMeNet | mNODE | VGPR | DMoVGPE |
| --- | --- | --- | --- | --- | --- | --- | --- | --- | --- | --- | --- | --- | --- | --- |
| MFGM | 1.1s | 0.4s | 25m | 1.4s | 13.4s | 5.6m | 12s | 13m | 15s | 1.5s | 3.2h | 3.3h | 21s | 15.3m |
| ASD | 0.7s | 0.5s | 13m | 0.6s | 0.8s | 27s | 2.3s | 3.4m | 3.2s | 1.2s | 58m | 1.1h | 4.2s | 3.3m |
| INFANTS_DIABETES | 1.1s | 1s | 41m | 1.3s | 15.7s | 4.5m | 5.4s | 9.5m | 14s | 2s | 41m | 4h | 5.8s | 6m |
| PRETERMS | 2.4s | 1.8s | 44m | 3.7s | 32.8s | 2.9m | 8.6s | 23m | 50s | 2.6s | 1.3h | 2.2h | 7.2s | 9m |
| SINHA_CRC | 2.9s | 2.4s | 1.2h | 4.4s | 1.1h | 3.6m | 23s | 24m | 91s | 3s | 1.4h | 2.6h | 30s | 6.2m |
| ADENOMAS | 2.4s | 1.9s | 2.5h | 5.2s | 18m | 5.3m | 45s | 21m | 84s | 2.6s | 4.5h | 4.7h | 1.8m | 13.3m |
| BIO_ML | 2.7s | 3.s | 1.5h | 4.6s | 4.3m | 4.7m | 37s | 46m | 66s | 2.6s | 2.1h | 3.1h | 18.1s | 6.6m |
| IBD_RELATIVES | 5.5s | 30.7s | 55m | 7.6s | 44.9s | 5.2m | 90s | 58m | 2m | 4.3s | 5.4h | 7.2h | 11.8s | 12m |
| IBS | 2s | 3.9s | 3.1h | 2.2s | 37.7s | 27m | 6m | 30m | 96s | 1.9s | 1h | 1.1h | 19.4s | 25.3m |
| CRC | 3s | 2m | 6h | 2.2s | 4.3m | 4h | 33m | 2.7h | 11m | 2.9s | 12.4h | 8.4h | 55.4s | 41.6m |
| GC | 1.8s | 59s | 8.3h | 2.3s | 60.8s | 1.4m | 13m | 21m | 6m | 2.4s | 12.6h | 8.9h | 6.3m | 7.8m |
| ESRD | 2.6s | 2.8m | 7h | 4.3s | 20m | 35m | 1.9h | 2h | 1h | 4.5s | 11.3h | 7.7h | 16.9m | 18.7m |
| IBD | 45s | 2.7h | 15h | 41s | 3.5h | 14.3h | 1.2d | 1.6d | 1.4d | 25.3s | 20h | 15.6h | 45.7m | 1.5h |
| IBDMDB | 9m | 1.8d | 3.4d | 7m | / | / | 4.2d | / | / | 4.7m | 10.2d | 1.9d | 7h | 15.4h |

The training times in Table 3 highlight the computational efficiency of DMoVGPE compared to other methods. While DMoVGPE requires more training time than VGPR due to its gating mechanism and joint optimization process, it is significantly faster than MiMeNet and mNODE on most datasets. For example, on the CRC dataset, DMoVGPE takes 41.6 minutes, while MiMeNet and mNODE require 12.4 hours and 8.4 hours, respectively. Similarly, on the ESRD dataset, DMoVGPE completes training in 18.7 minutes, compared to MiMeNet's 11.3 hours and mNODE's 7.7 hours. The longer training times of MiMeNet and mNODE are primarily due to their computational complexity: MiMeNet requires extensive hyperparameter tuning, while mNODE involves solving ordinary differential equations (ODEs), which is computationally intensive. Additionally, MelonnPan's training time is prolonged due to its reliance on grid search to identify optimal hyperparameters. In contrast, DMoVGPE achieves a balance between computational efficiency and model performance, making it a more practical choice for large-scale datasets. In terms of resource requirements, DMoVGPE's use of multiple epochs for training ensures better convergence and model performance, but it also increases the computational cost compared to methods like VGPR, which require only a single inference step. However, DMoVGPE is optimized to leverage both CPU and GPU resources efficiently, whereas methods like VGPR are primarily CPU-bound and do not scale well for large datasets. This efficient resource utilization, combined with its ability to model complex data distributions, makes DMoVGPE a more practical and effective choice for real-world applications.

In summary, while DMoVGPE has a higher computational complexity than VGPR due to its gating mechanism and joint optimization process, it achieves a better balance between computational efficiency and model performance. For large-scale and complex datasets, DMoVGPE is a more practical and effective choice than VGPR and other baseline methods. The detailed computational cost comparisons provided in this appendix further support the efficiency of DMoVGPE.

Reference

1. Haghikia A, Li XS, Liman TG, Bledau N, Schmidt D, Zimmermann F, Kränkel N, Widera C, Sonnenschein K, Haghikia A: **Gut microbiota–dependent trimethylamine N-oxide predicts risk of cardiovascular events in patients with stroke and is related to proinflammatory monocytes**. *Arteriosclerosis, thrombosis, and vascular biology* 2018, **38**(9):2225-2235.

2. Ratajczak W, Rył A, Mizerski A, Walczakiewicz K, Sipak O, Laszczyńska M: **Immunomodulatory potential of gut microbiome-derived short-chain fatty acids (SCFAs)**. *Acta Biochimica Polonica* 2019, **66**(1):1-12.

3. van der Hee B, Wells JM: **Microbial regulation of host physiology by short-chain fatty acids**. *Trends in Microbiology* 2021, **29**(8):700-712.

4. Postler TS, Ghosh S: **Understanding the holobiont: how microbial metabolites affect human health and shape the immune system**. *Cell metabolism* 2017, **26**(1):110-130.

5. Rahman S, O’connor AL, Becker SL, Patel RK, Martindale RG, Tsikitis VL: **Gut microbial metabolites and its impact on human health**. *Annals of Gastroenterology* 2023, **36**(4):360.

6. Jacob M, Lopata AL, Dasouki M, Abdel Rahman AM: **Metabolomics toward personalized medicine**. *Mass spectrometry reviews* 2019, **38**(3):221-238.

7. He X, Parenti M, Grip T, Lönnerdal B, Timby N, Domellöf M, Hernell O, Slupsky CMJSr: **Fecal microbiome and metabolome of infants fed bovine MFGM supplemented formula or standard formula with breast-fed infants as reference: a randomized controlled trial**. *Scientific reports* 2019, **9**(1):11589.

8. Kang D-W, Ilhan ZE, Isern NG, Hoyt DW, Howsmon DP, Shaffer M, Lozupone CA, Hahn J, Adams JB, Krajmalnik-Brown RJA: **Differences in fecal microbial metabolites and microbiota of children with autism spectrum disorders**. *Anaerobe* 2018, **49**:121-131.

9. Kostic AD, Gevers D, Siljander H, Vatanen T, Hyötyläinen T, Hämäläinen A-M, Peet A, Tillmann V, Pöhö P, Mattila IJCh *et al*: **The dynamics of the human infant gut microbiome in development and in progression toward type 1 diabetes**. *Cell host & microbe* 2015, **17**(2):260-273.

10. Wandro S, Osborne S, Enriquez C, Bixby C, Arrieta A, Whiteson KJM: **The microbiome and metabolome of preterm infant stool are personalized and not driven by health outcomes, including necrotizing enterocolitis and late-onset sepsis**. *Msphere* 2018, **3**(3):10.1128/msphere. 00104-00118.

11. Sinha R, Ahn J, Sampson JN, Shi J, Yu G, Xiong X, Hayes RB, Goedert JJJPo: **Fecal microbiota, fecal metabolome, and colorectal cancer interrelations**. *PloS one* 2016, **11**(3):e0152126.

12. Kim M, Vogtmann E, Ahlquist DA, Devens ME, Kisiel JB, Taylor WR, White BA, Hale VL, Sung J, Chia NJM: **Fecal metabolomic signatures in colorectal adenoma patients are associated with gut microbiota and early events of colorectal cancer pathogenesis**. *MBio* 2020, **11**(1):10.1128/mbio. 03186-03119.

13. Poyet M, Groussin M, Gibbons SM, Avila-Pacheco J, Jiang X, Kearney SM, Perrotta AsR, Berdy B, Zhao S, Lieberman TJNm: **A library of human gut bacterial isolates paired with longitudinal multiomics data enables mechanistic microbiome research**. *Nature medicine* 2019, **25**(9):1442-1452.

14. Jacobs JP, Goudarzi M, Singh N, Tong M, McHardy IH, Ruegger P, Asadourian M, Moon B-H, Ayson A, Borneman JJC *et al*: **A disease-associated microbial and metabolomics state in relatives of pediatric inflammatory bowel disease patients**. *Cellular and molecular gastroenterology and hepatology* 2016, **2**(6):750-766.

15. Mars RA, Yang Y, Ward T, Houtti M, Priya S, Lekatz HR, Tang X, Sun Z, Kalari KR, Korem TJC: **Longitudinal multi-omics reveals subset-specific mechanisms underlying irritable bowel syndrome**. *Cell* 2020, **182**(6):1460-1473. e1417.

16. Yachida S, Mizutani S, Shiroma H, Shiba S, Nakajima T, Sakamoto T, Watanabe H, Masuda K, Nishimoto Y, Kubo MJNm: **Metagenomic and metabolomic analyses reveal distinct stage-specific phenotypes of the gut microbiota in colorectal cancer**. *Nature medicine* 2019, **25**(6):968-976.

17. Erawijantari PP, Mizutani S, Shiroma H, Shiba S, Nakajima T, Sakamoto T, Saito Y, Fukuda S, Yachida S, Yamada TJG: **Influence of gastrectomy for gastric cancer treatment on faecal microbiome and metabolome profiles**. *Gut* 2020, **69**(8):1404-1415.

18. Wang X, Yang S, Li S, Zhao L, Hao Y, Qin J, Zhang L, Zhang C, Bian W, Zuo LJG: **Aberrant gut microbiota alters host metabolome and impacts renal failure in humans and rodents**. *Gut* 2020, **69**(12):2131-2142.

19. Franzosa EA, Sirota-Madi A, Avila-Pacheco J, Fornelos N, Haiser HJ, Reinker S, Vatanen T, Hall AB, Mallick H, McIver LJJNm: **Gut microbiome structure and metabolic activity in inflammatory bowel disease**. *Nature microbiology* 2019, **4**(2):293-305.

20. Lloyd-Price J, Arze C, Ananthakrishnan AN, Schirmer M, Avila-Pacheco J, Poon TW, Andrews E, Ajami NJ, Bonham KS, Brislawn CJJN: **Multi-omics of the gut microbial ecosystem in inflammatory bowel diseases**. *Nature* 2019, **569**(7758):655-662.
